# Sex/Gender Attribution: When the Penis Makes the Difference

**DOI:** 10.1007/s10508-021-02152-z

**Published:** 2021-11-15

**Authors:** Stefano Federici, Alessandro Lepri, Eleonora D’Urzo

**Affiliations:** 1 Department of Philosophy, Social and Human Sciences and Education, University of Perugia, Piazza G. Ermini 1, 06123 Perugia, Italy; 2 Myèsis, Research and Development Company, Rome, Italy

**Keywords:** Gender attribution, Sex attribution, Evolutionary psychology, Cognitive biases, Ethnomethodological approach, Gender identity

## Abstract

The present study aimed to replicate Kessler and McKenna’s (1978) ethnomethodological study that investigated how an individual attributes gender to a person. By administering figures depicted on overlays (Overlay Study), Kessler and McKenna found that the penis more than the vulva and the male sexual characteristics more than the female ones were significantly more salient in the gender attribution process. From all this, their adage is: “See someone as female only when you cannot see them as male.” Taking as a model Kessler and McKenna’s Overlay Study, we administered to 592 adults 120 new digital stimuli elaborated on realistic frontal images of human nudes to verify if the previously obtained results would be confirmed by using more realistic images. We found that the participants attributed male gender 86% of the time when the penis was shown, but only attributed female gender 67% of the time when the vulva was shown. All findings had strong statistical significance, confirming the findings of the Overlay Study that the penis makes the difference in gender recognition. Beyond an ethnomethodological approach, we have interpreted and discussed our results from the outlook of evolutionary and cognitive psychology and cognitive neuroscience, concluding that the cultural stereotypes and prejudices that affect gender attribution might not just be a mere cultural product, but rather the consequence of evolved cognitive biases.

**Supplementary Information:**

The online version contains supplementary material available at 10.1007/s10508-021-02152-z.

## Introduction

In this study, we often associate gender with sex/biological categories (male/female or penis/vulva), as we do in the title. We use the term gender (Bazarra-Fernandez, 2012; IPSOS, 2020; Krieger, 2003) because even sex variables are not understood in the objective, physical dimensions, but always subjective, as cognitive representations (Ito & Urland, 2003, 2005; Knutson et al., 2007). From this point of view of cognitive psychology, a primary sexual characteristic makes no difference, if not semantic, from a secondary one or from male, female, or unisex clothing (Carruthers et al., 2006; Tooby & Cosmides, 1992). All are ways of constructing reality (Olivetti Belardinelli, 1973). Our purpose is to investigate what influence each of these sexual characteristics has on cognitive processes of gender attribution (individual representations), not what determines an individual’s sex (biological). These cognitive processes and their products, the representations, are the outcome of determinants both internal to the human organism and external, often defined in the nature–culture relationship. This relationship will also be evident in the present study, where the representation of gender will be given by the outcome of sexual characteristics that will fluctuate from those more typically natural/biological to those more typically cultural, such as clothing.

Neuroscience studies have highlighted that our brains form us/them dichotomies (based on differences of social status, race, gender) with a staggering speed (Ito & Urland, 2003; Sapolsky, 2017). We know that a 50-ms exposure to the face of someone of another race activates the amygdala, while failing to activate the fusiform face area as much as same-race faces do—all within a few hundred milliseconds (Sapolsky, 2017). Similarly, the brain groups faces by gender or social status at roughly the same speed (Ito & Urland, 2003).

From an evolutionary perspective, it is understandable that there is the need for prompt recognition of us/them with respect to gender. We know that a fear emotional reaction is more likely to be conditioned by an angry male face than by a female face, and by an adult face than by a childish face (Dimberg & Öhman, 1996; Dimberg et al., 2000). Consistent with the evolutionary perspective, this mechanism has developed to avoid what is the greatest danger: an (angry) adult male. In addition, because people rely on simplifying the decision-making process, especially in cases of ambiguity, time pressure, or complexity (Kahneman, 2011; Korteling et al., 2018; Rachlin, 2003; Shah & Oppenheimer, 2008; Tversky & Kahneman, 1974), they revert to heuristic strategies. The human being must at all costs avoid confusing a male for a female, rather than the opposite. Lowering one’s defenses, because one has assumed to be in the presence of a female rather than a male, is more likely to cost one’s life compared to confusing a female with a male. For this reason, male sexual characteristics take on the function of attention targets in gender (signal) detection (Spackman, 1989). In case of ambiguity in the detection of gender cues, humans have had to avoid at all costs a false negative (detecting a female when it is male) because it is definitely riskier than a false positive (detecting a male when it is female). Furthermore, as the applied psychometry has demonstrated (Chadha, 2009), the false negative error (i.e., type I error rejecting the null hypothesis [H_0_] when it is true: detecting a female when there are male characteristics) and a false positive (i.e., type II error accepting H_0_ when it is false: detecting a male when there are no male characteristics) are mutually exclusive (inverse correlation). This type of correlation can only be justified if the two errors are affected by the same condition, that is, in the case of gender detection, the presence/absence of male sexual characteristics, but not in case the conditions were different, namely the presence/absence of male and female sexual characteristics.

As suggested by Navarrete et al. (2009), gender categorization might act as a heuristic cue for the potential for danger solely when the exemplars are male. Such an evolved cognitive mechanism occurs both in males and females. In fact, for all individuals (e.g., infants, females), the risk of socializing with a male is greater than with a female, because male individuals tend to be physically stronger and much more aggressive. In this view, to mistake a female when it is a male is potentially more dangerous than the opposite for human survival. Thereby, a subtraction is applied to the higher danger condition (male) to get to the lack-of-danger condition (non-male). In other words, to survive, it is much more convenient to make a wrong female than a male gender attribution. These errors of judgment are determined by cognitive mechanisms evolved by natural selection that “occurred despite the fact that subjects were encouraged to be accurate and were rewarded for the correct answers” (Tversky & Kahneman, 1974, p. 185). As an automatic evolved mechanism, the male *pre*-judice works as a cognitive constraint within which culture has been modeled and passed on by memes (Blackmore, 1999) of what we now call patriarchalism, androcentrism, and phallocentrism, by the means of imitation, education, religion, philosophy, and politics (Bem, 1993; Ranke-Heinemann, 1990). Although the functioning of the cognitive bias is “not attributable to motivational effects such as wishful thinking or the distortion of judgments by payoffs and penalties” (Tversky & Kahneman, 1974, p. 185), the cultural context and parental milieu nonetheless provide that ecological niche where such automatic behavior is reinforced and rewarded, that is, the motivating power of stereotypes, myths, beliefs, and ideologies (Federici et al., 2019).

Going back in history, even Freud assumed—what we have seen above was demonstrated by modern cognitive neuroscience—the existence of a universal, fast, and quite reliable cognitive mechanism of gender recognition. As he taught: “When you meet a human being the first distinction you make is ‘male or female?’ and you are accustomed to making the distinction with unhesitating certainty” (Freud, 1932/1964, p. 113). According to Freud (1925/1959), around the age of five, children become aware of their sexual/gender identity by becoming aware that they either possess or do not possess a penis. The recognition that one has or does not have a particular set of genitals, for Freud, is tantamount to recognizing the gender to whom they belong. “I have a penis” means “I am a boy,” and “I do not have a penis” means “I am a girl.” In this theory, the gender identity is a genital (penis-centered) identity.

Freud’s thought, therefore, assumes an apophatic way (i.e., a negative way, involving knowledge obtained by negation) to know the female identity. A child does not know what a female is, but they know what the penis does. A female also does not know what she is, because she is nothing but a human *non*-man or *not*-having-phallus. Literally, the female is but an absence (for a review on apophasis, see Zalta, 2020).

Through an ethnomethodological study, the feminist gender psychologists Kessler and McKenna investigated the apophatic gender construction; they found that penis equals male, but vulva does not equal female (Kessler & McKenna, 1978). When nude human figures were shown to participants having a vulva, the other male characteristics remained more powerful and dominant in affecting participant’s gender attribution. Of all the gender characteristics, the penis was the most “powerful” (salient). The results highlighted that “‘[m]ale’ gender attributions increased from 69 to 96% when a penis was added [to the stimulus figure] (p. 151).” When Kessler and McKenna then asked participants how they would change the nude figures to make them of opposite gender:

[i]n changing a male to a female[,] 38 percent of the participants mentioned removing the penis, but only one percent said that it was necessary to add a vagina. When changing a female to a male, the findings are reversed. Thirty-two percent of the participants said that a penis needed to be added to make a male but only one percent said that the vagina need be removed. (p. 153)

The salience of the penis was also observed by Thompson and Bentler (1971) in a study where nude dolls with various combinations of male and female gender characteristics were shown to adults and children. According to their findings, the masculine doll minus the penis was still seen as very male, but in its presence the feminine doll with a penis could not be seen as female.

Although the salience of the penis, and therefore its absence, remains decisive in gender attribution, the process of gender categorization does not only take place when information on genital characteristics is available. Even in the absence of this information, gender attribution is an automatic process that ends in milliseconds: The gender of a face is processed within 150 ms, after social dominance (40 ms) and race (100 ms) (Ito & Urland, 2003; Rule et al., 2012; Sapolsky, 2017).

Another study we think is worth considering in this regard, and that largely refers to the assumptions proposed by Kessler and McKenna (1978), is that carried out by Simpkins (2014). Using an ethnomethodological model, the study proposed to show participants images of parts of real human faces, created by photographing 28 people who varied by gender, ethnicity, and age, on the grounds that the face is “usually the first source of information available about a person” (Jackson, 1992, p. 3). The study concluded with a significant disproportion between the times in which the participants attributed male gender to the stimuli compared to times in which they attributed female gender to the stimuli, combined with an attribution of gender for 99% of the binary cases (male/female). It is interesting to note that even the interviewees who had identified themselves as having a non-binary gender made attributions related prevalently to male or female gender.

The most recent study that replicated Kessler and McKenna’s study used eye tracking on digital reproductions of original stimuli (Wenzlaff et al., 2018). Participants gazed longer at the head, chest, and genital areas of all the stimuli. Of note, they attributed female gender when a penis was present. It was associated with longer total dwell time than with male gender with a vulva shown. This is indicative of higher cognitive effort and more difficulty ignoring the penis as opposed to the vulva. Wenzlaf et al. concluded that the penis is a special cue in this attribution process.

The present study aims to replicate Kessler and McKenna’s (1978) ethnomethodological study with more realistic stimuli. In addition, if the results are confirmed, our study aims to go beyond an exclusively ethnomethodological interpretation of the data, using a more multidisciplinary approach, based on recent assumptions provided by cognitive neuroscience, cognitive psychology, and evolutionary psychology. Ethnomethodology is the study of how social order is produced in and through processes of social interaction (Garfinkel, 1967, 1974). According to the ethnomethodological perspective, “the gender is a social construction and a world of two ‘sexes’ is a result of the socially shared, taken-for-granted methods which members use to construct reality” (Kessler & McKenna, 1978, p. vii). As already defined above, our use of “gender” is not from an ethnomethodological perspective, but from a psychological one. We observe the male/female attribution as a product of a cognitive process, that is, as a phenomenological datum (gender), not a biological characteristic (sex). Like any cognitive process, this does not preclude that it may also be affected by social determinants, nor that the biological determinants were irrelevant.

In the following section, we will present the original experimental design (Overlay Study) of Kessler and McKenna (1978), from which we have developed our study.

### Background

**Fig. 1:**
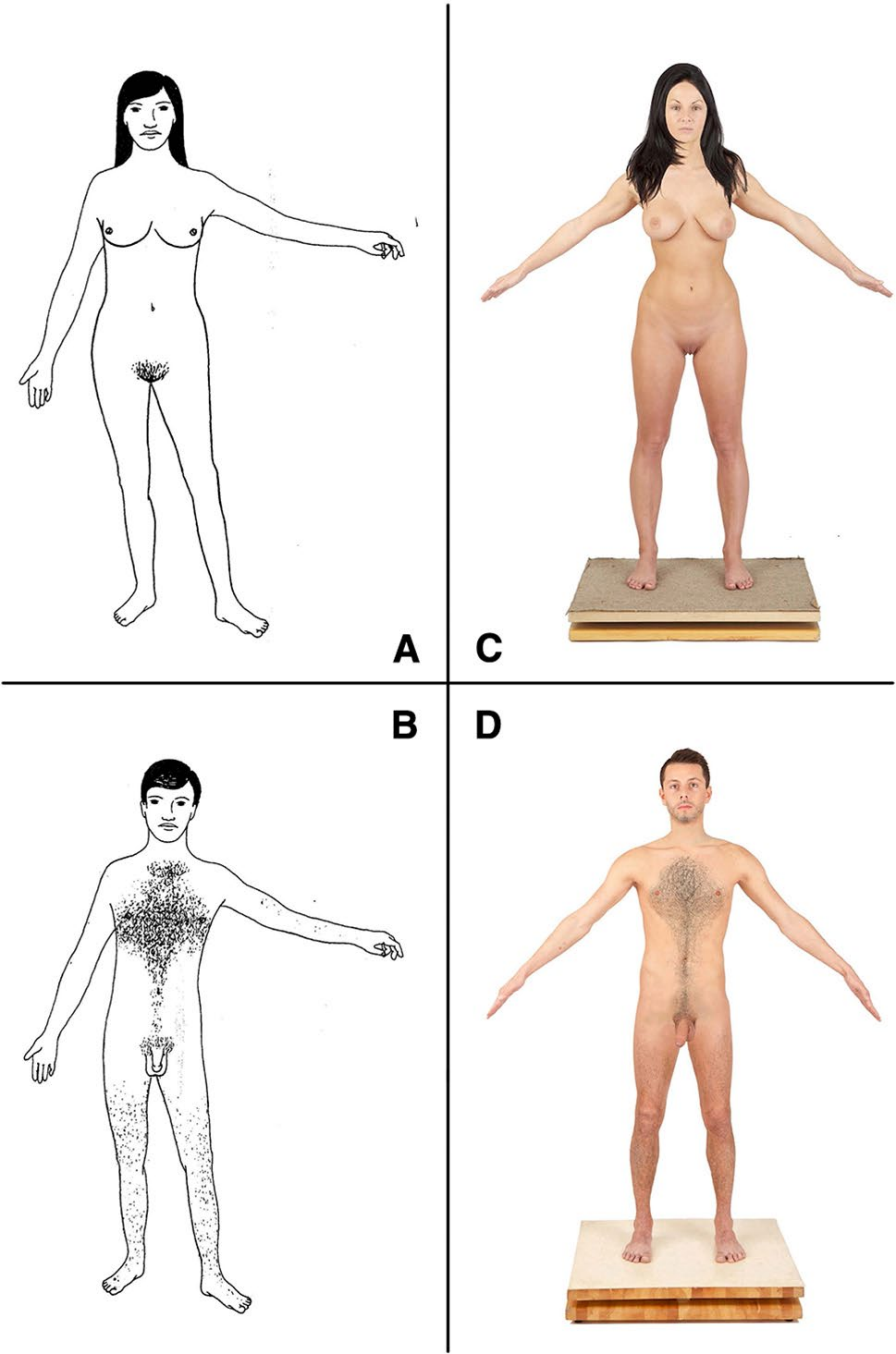
Male and female stimuli. A and B are, respectively, female and male images from the Overlay Study (Kessler & McKenna, 1978). C and D are, respectively, female and male human models selected for the Adult Gender Attribution Test. Images C and D where bought from the website www.3d.sk with a perpetual, non-exclusive, non-transferable worldwide license to use the content for permitted uses. All copyright and other intellectual property rights relating to the content are retained by 3D.sk

If human figures with ambiguous sexual characteristics (not immediately definable as male or female and therefore indicated by us as neutral) are shown, and if adult individuals are asked to determine such figures’ gender, according to common sense, one would expect that 50% of the participants would assign a “male” gender attribution to the figures, and 50% would provide a “female” gender attribution. But this does not happen, as already demonstrated by Kessler and McKenna (1978). They investigated gender attribution by showing to 960 adults in person 96 different figures depicted on overlays. Each image displayed a combination of nine human physical characteristics and two sets of clothing (Fig. 1a, b). The eleven overlays depicted people with: long hair, short hair, wide hips, narrow hips, breasts, a flat chest, body hair, penis, vulva, unisex t-shirt, or unisex pants. These overlays were superimposed one on top of the other. When the overlays were placed on a human figure with a face that was not gender-specific, the result was a drawing of a figure with various combinations of typically male and female physical gender characteristics. Therefore, the figures could represent a nude human being, one wearing or a unisex t-shirt or unisex pants, or a unisex t-shirt and unisex pants. Kessler and McKenna’s basic hypothesis was that a figure with more female than male characteristics would be regarded as female and vice versa: One with more male than female characteristics would have resulted in a male attribution. Kessler and McKenna also wondered how the presence or absence of certain stimuli, particularly the genitals, would affect the attribution of participants. The participants were asked three questions: (1) “Does this figure represent a male or a female?”; (2) “Using a scale from 1 to 7, where 1 means ‘not at all sure’ and 7 means ‘very sure,’ how sure are you of the answer you gave previously?”; and (3) “How would you change the figure so that it becomes something else?” On the basis of their findings, Kessler and McKenna (1978) affirmed to have found a “schema” among “members of the West reality” that leads to gender attribution: “See someone as female only when you cannot see them as male” (p. 158).

Therefore, taking into account the material produced for the Overlay Study, we created new stimuli by using photographs, not drawings, to verify whether the results obtained previously (Kessler & McKenna, 1978) would be confirmed when using more realistic images. According to evolutionary psychology, evolved psychological mechanisms would have evolved to solve specific adaptive problems, not generic logical problems. Therefore, they are triggered by competent environmental stimuli (Cosmides & Tooby, 1992). In accordance with this theory, we preferred to propose more realistic stimuli than those in the Overlay Study, to observe more ecological cognitive responses. This meant that, with the adoption of two human models, one male and one female, we no longer had a human figure with a face that was not gender-specific on which to run plastic overlays. Therefore, compared to the Overlay Study, we introduced two additional variables: male face and female face.

The process of displaying the stimuli is detailed below in the Method section (see Phase and Procedure).

### Hypotheses

1. Condition should influence gender attribution as follows (H1):
  - The mean of the male gender attributions is higher than the mean of the female gender attributions, *ceteris paribus*.
  - In the presence of primary sexual characteristics (penis, vulva), a significant increase in the attributions of the gender consistent with the external genitalia (penis → male, vulva → female), compared to the presence of secondary sexual characteristics (e.g., body hair or breast), is expected.
  - In the presence of primary sexual characteristics (penis, vulva), a significant increase in the attributions of gender consistent with the external genitalia (penis → male, vulva → female) is expected to occur, compared to when the genitals are covered (e.g., pants).
  - In stimuli where the penis is shown, the mean of male gender attributions is higher than when the vulva is shown.

These four hypotheses are elaborated on the base of Kessler and McKenna’s (1978) Overlay Study. For sexual characteristics, we mean them in the most common sense: *primary* are sex organs or those characteristics that are used to establish the sex of human beings at birth; secondary are those developed later in life, usually during puberty (Weininger, 2005). The expected effects should replicate those found in the Overlay Study even using more ecological stimuli.

2. Secondary sexual characteristics in the face should influence gender attribution as follows (H2):
  - We expect the mean of male gender attributions of faces with male secondary characteristics to be higher than the mean of female attributions of faces with female secondary characteristics.

A person’s face and genitals are sufficient on their own to predict a person’s gender (Jackson, 1992; Kessler & McKenna, 1978; Simpkins, 2014), though not with the same compelling strength. Specifically, Kessler and McKenna found that a stimulus with a face with male secondary characteristics and vulva, as well as a face with female secondary characteristics and penis, predicted a male attribution. This means that the salience of the penis succeeds in nullifying the presence of a female face and that the salience of a male face succeeds in nullifying the presence of a vulva. The most interesting data from Kessler and McKenna’s study is that the strength of male features (face or penis) can obliterate all other female cues (face or vulva).

3. The condition should influence confidence in gender attribution as follows (H3):
  - The confidence in the attribution of the male gender is expected to be significantly higher than that of the female gender, *ceteris paribus*.

As introduced above with regard to the signal detection theory, assuming that gender attribution is determined by discrimination of the presence/absence of male characteristics (target), we assume that the use of a false positive (attribution of male gender) is perceived as more reliable than a false negative (attribution of female gender) in ambiguous stimuli (Navarrete et al., 2009).

4. Condition should influence pleasantness of the stimulus as follows (H4):
  - The degree of pleasantness of the stimuli is significantly lower in the co-presence of male and female sexual characteristics.

In stimuli where both male and female sexual characteristics are present (e.g., narrow hips, body hair, breast, and vulva), we assume that the pleasantness of the image shown as a stimulus could be influenced by implicit (Kanamori et al., 2020) and explicit (Hill & Willoughby, 2005) transphobic responses.

## Method

### Study Design

This was an observational, quasi-experiment—as we did not perform any manipulation of the independent variables and there is no control group, and group assignment was based on characteristics that participants possess in nature (e.g., sex assigned at birth)—non-interventional, cross-sectional, and between-subjects study.

### Materials and Apparatus

#### Sociodemographic Questionnaire

This digital document was developed ad hoc to collect data on participants’ age, sex (as assigned at birth: male/female), gender identity (“I see/define myself a man”; “I see/define myself a woman”; plus Facebook’s 56 custom gender options for users who do not identify simply as “man” or “woman”), sexual orientation (Kinsey scale, see below), education, citizenship, religious beliefs, and political orientation.

#### Kinsey Scale

This is a classification system for sexual orientation in human beings (Kinsey et al., 1948), also called the Heterosexual–Homosexual Rating Scale. It ranges from 0 to 6, with 0 indicating exclusively heterosexual/opposite sex behavior or attraction and 6 indicating exclusively homosexual/same-sex behavior or attraction. Ratings 1–5 are for those who report varying levels of attraction or sexual activity with either sex. The additional category X, which the original Kinsey Report (Kinsey et al., 1948) included to designate no sociosexual contacts or reactions, was not utilized in administering the questionnaire.

#### Adult Gender Attribution Test

**Fig. 2:**
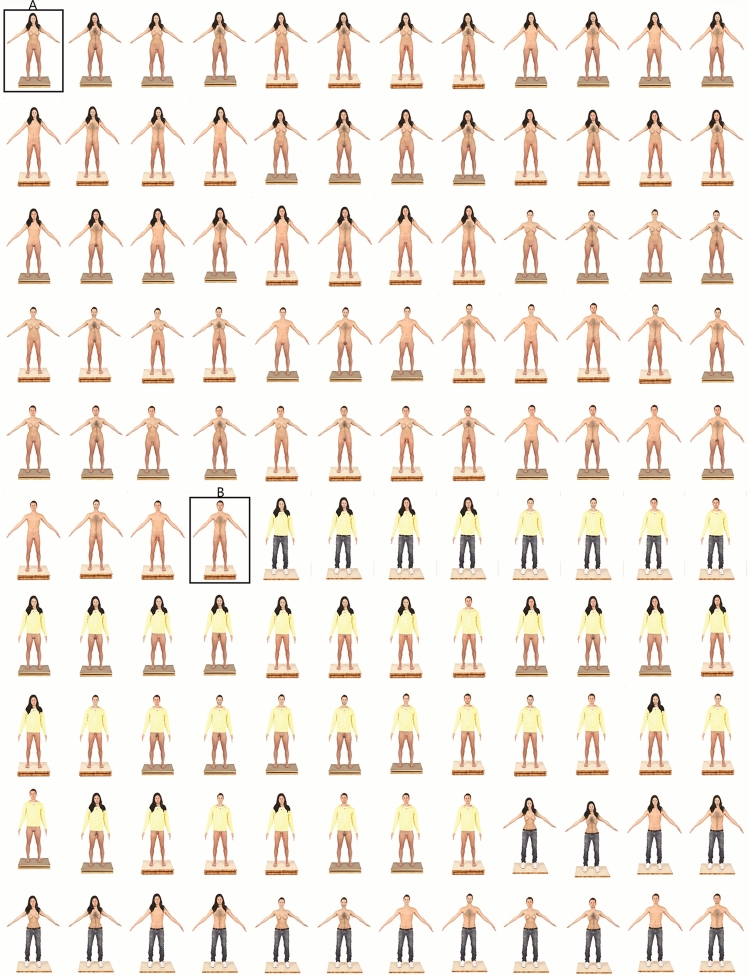
The 120 stimuli of the Adult Gender Attribution Test. The two framed stimuli, A (female) and B (male), are the original images bought from the website www.3d.sk with a perpetual, non-exclusive, non-transferable worldwide license to use the content for permitted uses. All copyright and other intellectual property rights relating to the content are retained by 3D.sk. The 118 unframed stimuli have been created through the software Adobe Photoshop 14 (see also Supplementary material)

This test was designed by the authors (SF and AL); it includes 120 images (Fig. 2; see also Supplementary material, Table S1) of frontal human nudes (masculine and feminine) created by combining parts of two original photographs of one male and one female model (see below, “Pre-experimental Phase”). For each stimulus presented through an Internet platform (www.qualtrics.com), the participants were asked to assign the male or the female gender (“According to you, is the subject in the picture male or female?”) and to indicate on two different 7-point Likert-type scales the degree of confidence with regard to the gender attributed to the stimulus (“How confident do you feel about the answer you just gave?”) and its pleasantness (“How pleasant is the picture you have just seen?”). Both scales were anchored by 1 (*not at all sure/pleasant*) and 7 (*very sure/pleasant*); higher scores indicate greater levels of confidence/pleasantness.

Access to the test was possible only through a dedicated computer. The experimental test (Sociodemographic Questionnaire and Adult Gender Attribution Test) was administered in Italian through the Internet platform www.qualtrics.com, after entering a password that only the investigators had.

### Phases and Procedure

#### Pre-experimental Phase

This phase was dedicated to selecting two images of human nudes, male and female, on which to create the experimental stimuli. Twenty photographs were bought from the website www.3d.sk; all had the same digital visual characteristics (light, exposure time, distance from the lens, resolution, etc.) across male and female models; all were shown in full nude in the frontal position, aged between 21 and 30 years, white Caucasian, without visible marks on the body such as scars or tattoos (Supplementary material, Table S12). The 20 images were submitted to a sample of 200 adults (*n* = 109 males), in random order, through an Internet platform (www.surveygizmo.com). For each stimulus, the participants were asked to assign the male or the female gender (“According to you, is the subject in the picture male or female?”). Depending on whether participants answered male or female, they were asked how masculine or feminine they thought the subject in the photo was by indicating on a 7-point Likert-type scale the degree of the masculinity or femininity (“How masculine/feminine does the subject in the previous photo look to you?”). The scale was anchored by 1 (*not at all masculine/feminine*) and 7 (*very masculine/feminine*); higher scores indicated greater levels of masculine/feminine. The two images of male and female nudes that received the highest score as representing male (Fig. 1d) or female (Fig. 1c) models, respectively, were chosen as “original” images on which to perform body morphing through the software Adobe Photoshop 14. According to the Overlay Study (Kessler & McKenna, 1978), from the combination of the six male (short hair, male face, flat chest, narrow hips, penis, and body hair) and six female (long hair, female face, breast, wide hips, vulva, and no body hair) variables, plus two pieces of clothing (pants and t-shirt), 120 stimuli were created depicting frontal human nude shots (Supplementary material). For primary sexual characteristics, we mean the variables of external genitalia (penis and vulva); for secondary/gender-linked sexual characteristics, we mean short/long hair, male/female face, flat chest, breasts, narrow/wide hips, and body/no body hair. The images ranged from the original photograph of the male model (all male variables; Fig. 1d) to the original photograph of the female model (all female variables; Fig. 1c) to three variables both male and female with or without clothes (Fig. 2; see also in Supplementary material the figures coded 1_F1 and 64_M1). To proceed with data analysis and interpretation, we coded with the letter “M” (47 male stimuli) those stimuli with a majority of male variables (> 3), with “F” (47 female stimuli) those with a majority of female variables (> 3), and with “N” (26 neutral gender stimuli) those with a balanced co-presence of male (= 3) and female (= 3) variables (Supplementary material, Tables S1 and S2). These codes remained completely unknown to the participants and related only to the research (null) hypothesis, namely that all sexual variables have equal salience. Therefore, M = male stimulus, F = female stimulus, and N = gender neutral stimulus do not refer to the perception of the figures nor to an evaluation of a biological sex of the figure represented in the stimulus. M, F, and N refer only to the quantitative distribution of the variables in each stimulus, namely as the figures were depicted and manipulated. As Kessler and McKenna (1978) used a stylized drawing of a neutral human face, their stimuli resulted in 96 combinations. In comparison, we have 120 stimuli in our study, because adopting real human faces imposed two other variables: male face and female face (Fig. 2).

#### Participants in the Pre-experimental Phase

Participants were recruited through social networks (Facebook, Instagram, and Twitter). The test was administered to 200 adults (gender assigned at birth: female = 91; 45.5%). The median age was 25 years (23%; Min = 18; Max = 65). One hundred and ninety-five participants were born in Italy (97.5%), four in Albania (2.0%), and one in Afghanistan (0.5%). The majority were undergraduate (81.5%), and 8.5% were graduate students. According to the Kinsey scale, 181 (90.5%) affirmed that they were exclusively heterosexual, four (2.0%) were exclusively homosexual, and 15 (7.5%) were bisexual.

#### Experimental Phase

The task was administered individually to each participant in rooms located at the University of Perugia’s campus, set up with desks and chairs, with personal computers (PCs) dedicated exclusively to experimentation and protected by a password. Before the PC was switched on, the participant was given an information sheet (describing the procedure for administering the test and advising the participants that they would be exposed to full nude photos) and an informed consent form (explaining how data would be processed by the investigators) in paper format to be signed. After the participant signed the documents, the computer was switched on and the www.qualtrics.com platform was started. At this point, the investigator left the room and advised the participant that they could call them back at any time by ringing a reception bell placed on the desk. The Internet platform then provided the participant with the Sociodemographic Questionnaire and, afterward, the 120 stimuli of the Adult Gender Attribution Test in random order. The online administration, in total, took about 40 min to complete.

#### Participants in the Experimental Phase

The participants were recruited by trained graduate students in psychology at the University of Perugia’s campus. All those who were available, both students and non-students (e.g., university students, employees, and visitors), if they were of age, were invited to participate in the experiment. No other (conscious) selection criteria were applied in the selection of the participants other than age (≥ 18 years).

The test was administered to 592 adults (gender assigned at birth: female = 68.4%). The median age was 19 years (23%; Min = 18; Max = 90; see Table 1). The majority were undergraduates (62.2%; female = 63.2%). With regard to political affiliation, the majority claimed to not identify with any political orientation (33.4%; female = 32.8%) or to not know (17.1%; female = 19.5%). Among the participants who expressed a political orientation, the majority declared a left-wing one (23.5%; female = 23.5%). The two major and almost exclusive groupings in which respondents placed themselves with respect to religious affiliation were Catholicism (51%; female = 54.1%) and no religion (42.4%; female = 38.8%; see Table 1).

**Table 1:** Descriptive statistics of sociodemographic variables and biological sex

Item	*n* (%)	Males (%)	Females (%)	*χ*^2^ (df)	*p*
Participants	592 (100)	31.6	68.4	80.28 (1)	< .001
*Level of education*				5.71 (5)	.335
Primary school diploma	10 (1.7)	3.2	1.0		
A few years of secondary school (high school)	15 (2.5)	3.2	2.2		
Secondary school diploma (higher)	343 (57.9)	53.5	60.0		
A few years of university (up to the 3-year degree)	168 (28.4)	30.5	27.4		
Master’s degree	45 (7.6)	7.5	7.7		
Post graduate master’s degree/Ph.D	11 (1.9)	2.1	1.7		
*Political orientation*				6.53 (6)	.367
Right wing	32 (5.4)	6.4	4.9		
Center-right wing	43 (7.3)	8.6	6.7		
Center	25 (4.2)	4.3	4.2		
Center-left wing	54 (9.1)	10.7	8.4		
Left wing	139 (23.5)	23.5	23.5		
None	198 (33.4)	34.8	32.8		
Don’t know	101 (17.1)	11.8	19.5		
*Religion*				10.54 (6)	.104
Catholic Christian	302 (51.0)	44.4	54.1		
Orthodox Christian	9 (1.5)	1.6	1.5		
Evangelical/reformated Christian	3 (0.5)		0.7		
Islamic	4 (0.7)		1.0		
Buddhist	2 (0.3)		0.5		
No religion	251 (42.4)	50.3	38.8		
Other	21 (3.5)	3.7	3.5		

Of the participants, 97.1% identified themselves in the man/woman binary gender identity among the 58 gender identity options (Table 2). According to the Kinsey scale, 495 (83.6%) affirmed that they were exclusively heterosexual, 6 (1.0%) were exclusively homosexual, and 18 (3.0%) were bisexual.

**Table 2:** Frequencies of gender identity

Item	Frequencies	Percent	Cumulative percent
*I see/define myself*			
Man	189	31.9	31.9
Woman	386	65.2	97.1
Agender	2	0.3	97.5
Androgynous	1	0.2	97.6
Bigender	2	0.3	98
Cisgender	1	0.2	98.1
Cis female	1	0.2	98.3
Cis male	3	0.5	98.8
Cisgender man	1	0.2	99
Female to male	1	0.2	99.2
Gender fluid	2	0.3	99.5
Gender nonconforming	1	0.2	99.7
Gender questioning	2	0.3	100
Total	592	100	

*Note.* Out of the 58 options, only those that were chosen at least once are shown

### Data Analysis

Descriptive statistics were conducted to explore the features of the sample. Inferential statistics (multivariate and univariate analysis of variance [ANOVA]) were used to determine the effect of the independent variables—extracted through the Sociodemographic Questionnaire (gender identity, level of education, political orientation, religious orientation) and the Kinsey scale (sexual orientation)—on the basis of the gender attribution. *t* tests for unpaired samples were calculated to compare the means of the responses of males and females to the Adult Gender Attribution Test. Chi-square tests were used to explore the hypothesis that sociodemographic variables are independent of biological sex.

To determine whether stimuli containing genital features (i.e., genitals uncovered, genitals covered, secondary/gender-linked sexual characteristics, and faces with male or female characteristics) significantly affect gender attribution in a congruent way (i.e., penis → male, vulva → female), gender attributions (male vs. female) were subjected to a mixed logistic regression (core odds ratio [cOR]) with fixed effects for the following categorical variables:

1. Congruence gender attribution: We assumed that the pictures with many female characteristics (female face, long hair, wide hips, breasts, no body hair, vulva; *n* = 50 stimuli) would be seen as female and those with many male characteristics (male face, short hair, narrow hips, flat chest, body hair, penis; *n* = 50 stimuli) would be seen as male; in other words, pictures with > 3 characteristics representative/evocative of that gender. The remaining 20 stimuli, where a balanced co-presence of male and female characteristics and without clothing were mixed, were excluded from this analysis.
2. Salience of primary sexual characteristics, namely a penis and a vulva (*n* = 48 stimuli with a penis vs. *n* = 48 stimuli with a vulva).
3. Salience of primary sexual characteristics compared to all other sexual characteristics—the pictures with uncovered genitals (*n* = 96) showed a primary sexual characteristic (penis or vulva) and those where the model wore pants, that is, with covered genitals (*n* = 16), showed all other sexual characteristics.
4. Salience of primary sexual characteristics compared to secondary/gender-linked sexual characteristics only—all the pictures with uncovered genitals (*n* = 96) and pictures where the model wore pants or t-shirt and pants, that is, with covered genitals (*n* = 24), showed secondary/gender-linked sexual characteristics.

## Results

All research data are available in a public repository (Federici et al., 2020).

A *t*-test was run where the participant’s gender in a binary mode (man/woman) was the independent variable and the dependent variables were the means of the responses to the Adult Gender Attribution Test. There were no differences between the groups (see the Supplementary material, Table S3).

One-way between-subjects ANOVAs were conducted to compare the effect of the independent variables—extracted through the Sociodemographic Questionnaire (gender identity, level of education, political orientation, and religion orientation) and the Kinsey scale (sexual orientation)—on the basis of the gender attribution, the confidence scale, and the pleasantness scale (see the Supplementary material, Tables S4–S8). Because none of the ANOVAs revealed a significant effect, we do not report results of any post hoc test.

We also evaluated the measure’s reliability regarding internal consistency calculated with Cronbach’s alpha. This coefficient was good (α = 0.86) for the gender attribution (male/female) based on the 120 stimuli and excellent (α = 0.99) for both the confidence scale and the pleasantness scale (see the Supplementary material, Tables S9–S11).

### The Effect of Male Sexual Characteristics on Gender Attribution (H1a)

Descriptive analysis showed that the mean of male gender attribution (H1.a) was 60.1%, *χ*^2^(1, *N* = 592) = 2902.74, *p* < 0.001.

A significant mixed logistic regression, *F*(1, 42,002) = 13,237.90, *p* < 0.001, where the dependent variable was gender attribution (male is coded as 1) and the independent variable was the presence of primary characters in the picture (0 is equal to the vulva shown in the pictures), indicates that, when the vulva is displayed, male attribution is 192 times more likely than female attribution when the penis is shown (cOR 191.56, 95% CI [175.19, 209.54], *p* < 0.001; see the Supplementary material).

### The Effect of Primary Sexual Characteristics on Gender Attribution (H1b)

In the presence of primary sexual characteristics (penis or vulva), we expected a significant increase in the attributions of gender consistent with the external genitalia (penis → male, vulva → female) compared to the presence of secondary sexual characteristics (e.g., body hair or breasts). In other words, we assume that the presence or absence of genitals (stimuli with uncovered genitalia) affects the participants’ perceptions of other physical characteristics more than the sum of the male or female variables that depict each stimulus.

The descriptive analysis of frequencies confirms this hypothesis: When primary genitals were shown, gender consistent with the external genitalia was around 75.7% for males and 70% for females, *χ*^2^(1, *N* = 592) = 174.81, *p* < 0.001. The percentage of attributions consistent with the external genitalia (penis and vulva), in the case of the presence of secondary/gender-linked sexual characteristics (e.g., body hair or breasts), fell to 51%, χ^*2*^(1, *N* = 592) = 60.40, *p* < 0.001. A significant mixed logistic regression, *F*(1, 63,306) = 77.59, *p* < 0.001, where the dependent variable was congruence (i.e., gender attribution in line with genitals, coded as 1 = congruent) and the independent variable is the presence of primary characters in the picture (1 is equal to genitals shown in the pictures), indicates that when primary genitals are displayed, attribution is congruent with the stimulus gender about two times more than when only secondary characteristics are shown (cOR 1.65, 95% CI [1.47, 1.85], *p* < 0.001; see the Supplementary material).

### The Effect of Covered vs. Uncovered External Genitalia on Gender Attribution (H1c)

In the presence of primary sexual characteristics (penis or vulva), we expected a significant increase in the attributions of gender consistent with the external genitalia (penis → male, vulva → female) compared to when the genitals were covered (e.g., pants).

When genitals were covered, the gender attribution was only congruent to genitals 47.4% of the time, *χ*^2^(1, *N* = 592) = 484.29, *p* < 0.001.

A significant mixed logistic regression, *F*(1, 68,042) = 157.82, *p* < 0.001, where the dependent variable was congruence with genitals (1 is congruent and served as the reference category) and the independent variable was covered genitals (where 1 represents uncovered genitals), indicates that when external genitalia were shown, attribution was congruent with the depicted stimulus genitals around two times more than when they were covered (cOR = 1.88, 95% CI [1.71, 2.08], *p* < 0.001; see the Supplementary material).

### The Penis Effect on Gender Attribution (H1d)

When the penis was uncovered (human figures without pants), 86.3% of participants, *χ*^2^(1, *N* = 592) = 15,966.15, *p* < 0.001, attributed male gender to the pictures; when the vulva was exposed, only 66.9% of participants attributed female gender. A significant mixed logistic regression, *F*(1, 42,002) = 1137.54, *p* < 0.001, where the dependent variable was congruence with genitals (congruence was coded with 1) and independent variable primary sexual characters uncovered in the picture (penis was coded as 1), indicates that when the penis was exposed in the picture, the participants attributed male gender about five times more often than female gender when the vulva was exposed (cOR 4.74, 95% CI [4.33, 5.19], *p* < 0.001; see the Supplementary material).

### The Face Effect on Gender Attribution (H2)

Pictures (*n* = 15) with a female face and one to four secondary female characteristics (long hair, wide hips, breasts, and no-body-hair) in 66.2% of instances received female gender attribution, while stimuli (*n* = 15) with a male face and one to four secondary male characteristics (short hair, narrow hips, flat chest, body hair) in 74.2% of occurrences received male attribution, *χ*^2^(1, *N* = 592) = 2923.02, *p* < 0.001. A significant mixed logistic regression, *F*(1, 17,748) = 5849.60, *p* < 0.001, where the dependent variable was gender attribution (female coded with 1, male coded with 0) and independent variable was face effect (female face coded as 1, male face coded as 2), indicates that when female faces were displayed, gender attribution was congruent with female gender around 1000 times less than when a male face was depicted (cOR 0.001, 95% CI [0.00, 0.002], *p* < 0.001; see the Supplementary material).

### Confidence in Gender Attribution (H3)

If we consider the conditions where the penis was exposed, 27.1% of the participants gave a certainty score of 7, indicating they had no doubt about the picture’s gender. Conversely, when the vulva was exposed, 19.3% of the participants gave a certainty score of 7, *χ*^2^(1, *N* = 592) = 454, *p* < 0.001. When participants attributed female gender, 79.2% of participants declared they were uncertain (scores 1–6), but when participants attributed male gender, 73.4% of participants indicated uncertainty, *χ*^2^(1, *N* = 592) = 311.1, *p* < 0.001.

### Pleasantness in the Co-presence of Male and Female Sexual Characteristics (H4)

Of the participants, 41.5% found the pictures with the 20 neutral gender stimuli (balanced co-presence of male and female variables and no clothing; Supplementary material, Table S2) very unpleasant (1). By contrast, 32.3% of the participants found the picture very unpleasant when it had unbalanced sexual variables, *χ*^2^(6, *N* = 592) = 670.25, *p* < 0.001 (*n* = 50 with a prevalence of variables extracted from the original male image: *n* = 50 with a prevalence of variables extracted from the original female image). When there were neutral stimuli, 2.4% of the participants found the image complete pleasant (1), which is lower than around 5.9% of participants who found the image completely pleasant when it had unbalanced sexual variables, *χ*^2^(1, *N* = 592) = 242.07, *p* < 0.001.

## Discussion

We aimed to replicate Kessler and McKenna’s (1978) Overlay Study, which administered stylized drawings of the human body, by using realistic images taken from pictures of human models. We expected to find confirmation that primary sexual characteristics (genitals) would determine gender attribution (male/female) more than secondary/gender-linked sexual characteristics, and that male sexual characteristics would determine gender attribution more than female sexual characteristics, with a significantly stronger effect of the penis compared to the vulva, *ceteris paribus*. The results have disconfirmed the null hypothesis and substantially reconfirmed the results obtained in the previous Overlay Study. When the penis was shown in a picture, the participants attributed male gender 86% of the times when the penis was shown, but only 67% attributed female gender when the vulva was shown. In other words, female attribution when the vulva appeared in a human picture was about 1:200 (female/male) compared with male attribution when the human body showed a penis. Furthermore, the participants attributed male gender to neutral stimuli (3 male and 3 female variables) five times more often when the penis was displayed than when the vulva was shown. All findings had a strong statistical significance, leading us to substantiate the Kessler and McKenna’s (1978) adage, “See someone as female only when you cannot see them as male” (p. 158). Female gender attribution appears to be triggered only when every other male cue has been excluded. In other words, gender cues are neither equally powerful nor salient. Therefore, all other things being constant, a female cue is recognized as such only in the absence of male cues. Whereas a male gender cue most likely equals male, a female cue equals female with much less probability. In this sense, we have defined above the Freudian and ethnomethodological attribution of female gender as an apophatic process.

Unlike Kessler and McKenna’s ethnomethodological approach, which they used to explain the gender attribution as a purely cultural construction, we also use the interpretative model of evolutionary psychology, which has spread after the Overlay Study, mainly by Cosmides and Tooby (Cosmides, 1989; Cosmides & Tooby, 1992; Tooby & Cosmides, 1992). Evolutionary psychology overcomes any dichotomy between nature and nurture or biological and cultural. Psychological/cognitive mechanisms/processes and cultural products co-evolve, so that environment forms; environment is necessary for the emergence and activation of each mechanism/process. Therefore, given that behavior requires evolved psychological mechanisms combined with environmental input in a causal chain, beyond the ethnomethodological interpretive model that makes use of the cultural construction of gender (patriarchal and phallocentric), we provide an explanation of our findings from an evolutionary psychology approach as cognitive adaptations that guide human behavior to adapt to the environment.

Seen from a less dichotomous and more dialogic and circular approach to interrelations between nature and culture, gender attribution is neither just pre-programmed nor simply derived from the social environment. The fact that, in a phallocentric culture, a penis makes somebody a male and not a female, as Freud’s psychological theory of human development also taught, does not negate the fact that these evolved cognitive adaptations were guided by an adapted mind (Tooby & Cosmides, 1992). There is no doubt that, in patriarchal cultures, the female role is derived from the space left free by the male role, though still under patriarchal control. So we can read biological cues as cultural: “the only sign of femaleness is an absence of male cues” (Kessler & McKenna, 1978, p. 152). However, this does not contradict that what culture has expressed, strengthened, sedimented, socially stratified, and handed down through cultural products and memes may have evolved from cognitive processes that have guaranteed human survival (Barkow, 1992; Buss, 2001; Carruthers et al., 2006; Ji & Yap, 2016; Lumsden & Wilson, 1981). In case of ambiguity or complexity in the detection of gender cues, a cognitive bias has saved humans from a risky encounter with an aggressive male (Dimberg & Öhman, 1996; Dimberg et al., 2000; Navarrete et al., 2009). This is consistent with our data about the ability to recognize a masculine face as a face of a man—this attribution is 1000 times more likely than attributing a feminine face to a woman—or about the certainty in gender attribution, according to which participants stated they were less certain when they had to attribute female gender to a picture (as opposed to male gender). This finding would also rule out the null hypothesis for the second assumption (H2). A male face is an excellent predictor of male gender attribution (Jackson, 1992; Simpkins, 2014) and, if associated with the penis, as previously found by Kessler and McKenna (1978), can overshadow all other female cues (face or vulva).

Unlike Kessler and McKenna’s study, the present investigation also examined the pleasantness of the *trans*-images, that is, those with a balanced co-presence of male (= 3) and female (= 3) variables (Supplementary material, Tables S1 and S2). Of the participants, 98% found unpleasant the 20 neutral gender pictures, significantly more than how they felt about other pictures with unbalanced gender sexual characteristics. This can be explained in several ways that are not necessarily mutually exclusive. These cross-sex/gender/clothing pictures, more than others, might recall the image of transsexual individuals, thus triggering the widespread sense of transphobia (Hill & Willoughby, 2005; Killermann, 2017). It is equally likely that the strong ambiguity of these pictures could have triggered the so-called uncanny valley phenomenon, that is, the emotional response to a humanoid whose resemblance to a human being is confusing (Mori, 1970). These 20 stimuli, more than others, confused respondents because they did not allow a clear dichotomous gender attribution (humanoid: human = picture with balanced co-presence of gender characteristics: picture with non-balanced co-presence of gender characteristics; MacDorman et al., 2009). In addition, processing facilitation increases positive affect (Winkielman & Cacioppo, 2001), also described as the pleasure of cognitive ease (Kahneman, 2011) or feeling of familiarity (de Vignemont, 2018). This uncanny valley/transphobia effect, caused by neutral stimuli, can also be explained using, once again, signal detection theory (Spackman, 1989). The perturbing effect (uncanny valley/transphobia) occurs when an individual cannot recur to a simplifying heuristic—to a rule of thumb, as (Kahneman, 2011) wrote—to make a difficult judgment about the gender of a person, due to the co-presence of salient male and female cues of sexual characteristics, for example, people with intersex or transsexual characteristics (in our study, the neutral stimuli).

### Characteristics of the Sample

Based on the results, the main sociodemographic variables of the participants, treated independently, did not significantly affect the responses on the Adult Gender Attribution Test. The sample was mostly young adults (about 19 years of age), certainly due to the fact that the recruitment took place on a university campus. Of the participants, 97% identified themselves in the male/female binary gender mode among Facebook’s 58 gender options. We have no official data on the transgender population in Italy: While we are writing this work, the first demographic survey is underway in Italy (see: www.studiopopolazionespot.it/). According to the demographic study on transgender and gender non-binary people in the United States by (Nolan et al., 2019), the range of estimated prevalence is 0.39–2.7%. Therefore, transgender non-binary people among the participants of this study (2.9%) seem to be slightly higher than the U.S. range. Less than 1% of males (sex assigned at birth) did not see/define themselves as a man, choosing another gender identity. By contrast, 3% of females did not feel like a woman; this was significantly different from males. The males had remained more “loyal” to their cisgender identity, that is, their sex assigned at birth aligns with their gender identity and gender expression (Killermann, 2017), compared with their female counterparts. Put another way, “I’m a male, so I’m a man.” This increased female *trans*-sexuality that appears in our sample is in line with what has already been found in several studies (Baumeister, 2000; Lehmiller, 2018): Women’s sexual behavior is more variable (flexible) than men’s over the course of their lives.

Regarding sexual orientation, our sample does not seem to match the survey data reported by the National Institute of Statistics (ISTAT, 2012) on the homosexual population. ISTAT found that about 2.4% of the Italian population declared themselves to be homosexual or bisexual and 77% heterosexual. Conversely, 84% of our participants affirmed themselves to be exclusively heterosexual and 4% homosexual or bisexual. The difference can be explained by the fact that the survey of sexual orientation was conducted with different metrics. In fact, ISTAT percentages refer to a breakdown of the population into categories that include both sexual orientation (hetero, gay, and bisexual) and gender identity (transgender and other identity).

Regarding religious affiliation, the Catholic affiliation of participants was lower (53%)—but higher than no religion (42%)—than the Italian average among a student population aged between 18 and 34 (respectively, about 61% and 36%), according to Ipsos Public Affairs (2017). Addressing a sexually explicit issue seems to attract more people without religious affiliation and with a left-wing political orientation (33%).

### Limits of the Study

We will discuss what we consider to be the most relevant limitation of the study below.

1. *Generalizability of the results* (Kukull & Ganguli, 2012). Although the research is based on a large number of participants, due to the characteristics of the sample (e.g., the prevalence of university students) and the recruitment context (i.e., a university campus), the results may not be generalizable, for example, to the Italian population.
2. *Social desirability* (Babbie, 2010). Whenever we ask people for information, they answer through a filter of what will make them look good. This is especially true when participants are asked to respond to extremely sensitive topics such as sexuality. For example, a respondent was convinced that the test was intended to measure attitudes toward transgender people—despite the fact that, to prevent any misunderstanding about the purposes of the research, the information sheet expressly stated that the study did not concern transphobia, “gender theories,” or queer theories. That person could have answered the question, “How pleasant is the picture you have just seen?” with a higher score of pleasantness, so as not to appear as a moralistic transphobic, intolerant person. This limit of explicit measures on sexuality (Baumeister, 2000) could be overcome by using procedures of implicit gender attribution (Wittenbrink & Schwarz, 2007), knowing that gender attribution is processed below the level of consciousness (subliminally; Ito & Urland, 2003; Sapolsky, 2017).
3. *Ecological experimental context*. Although the purpose of our study was to replicate the Overlay Study with more realistic stimuli, this does not mean that the environmental setting and nature of the stimuli were entirely ecological. For example, a more ecological gender-attribution process could have been observed in how an individual attributes the gender of a live person in their presence. Now, if it is true that gender attribution is processed by an evolved mental module to solve a specific adaptive problem (e.g., recognition of an aggressive male), the specificity of these cognitive modules is extremely dependent on the context (Fodor, 1983). As evolutionary psychology teaches us, an innate psychological mechanism is not equally effective in solving a logical abstraction of the same problem, for example, in solving the same problem through the solution of abstract syllogism (Cosmides, 1989; Cosmides & Tooby, 1992, 1994).
4. *Cross-cultural method*. The presented experimental design did not include a comparison between samples from different cultures. However, comparing different cultures would allow us to investigate whether the process of gender attribution can be ascribed more to human universals (Atran, 1998; Brown, 1991)—that is, to the effect of evolved psychological mechanisms—rather than to the influence of memes.
5. *Replication of the Overlay Study*. We decided to exclude in the Adult Gender Attribution Test one question that was included in the original study: “How would you change the figure to make it into the other gender?” (Kessler & McKenna, 1978, p. 146). This question should have been presented below each of the 120 stimuli and would have enabled us to analyze relevant qualitative information. Our choice was dictated by the fact that we did not want to increase the time needed to complete the test, especially because it was administered online (which would have compromised the reliability of the data we wanted to collect).

During the review process of the present study, one of the reviewers pointed out to us that we should have acknowledged in some way that sex is not binary, as follows:

Some people with XY chromosomes never develop a penis because they have an insensitivity to androgens. Some people are intersex. Some have too many or too few sex chromosomes. And as we learn more about the variations in the biology of people that impact their sex and gender identity it is important to understand sex/gender as cultural. If a penis is what makes someone male, then are sex reassignment surgeries to either add a penis (for transgender men) or remove a penis (for transgender women) more common than the addition or removal of a vulva?

We fully agree with the reviewer’s assertion that sex is not binary, and that reality (biology) is more complex than how psychological mechanisms, evolved to solve adaptive problems related to survival, lead us to think fast (about fast and slow thinking, see Kahneman, 2011). We feel differently about gender transition. Counterintuitively, in our view, a female-to-male transition is easier than a male-to-female transition for two patriarchal cultural reasons: (1) the male androcentric model is socially better defined and aspired to than the female model and thus easier to adopt; and (2) a male-to-female transition involves a more serious transgression of power hierarchies than vice versa and thus is socially more difficult to accept. However, this issue is far beyond the scope of this study and deserves empirical verification that this study does not provide.

### Conclusions

This study has confirmed what, as early as 1978, Kessler and McKenna found: The penis makes the difference in gender recognition. To put it more clearly, what guides an individual in gender attribution are typically two classes of variables: genitalia and face. But *ceteris paribus*, of both classes the male ones have a particular strength in orienting the gender attribution process. The penis, more than the vulva, the male face, more than the female face, and in general the male sexual characteristics more than the female ones, are significantly more salient in the gender attribution process. The data and theories presented in the current study guided us beyond Kessler and McKenna’s ethnomethodological approach. At the end of this study, we are inclined to think that Freud’s version of the one-sex model is not only a patriarchal phallocentric elevation of a “biological fact” into a cultural desideratum. It captures processes of psychological functioning, which reveal to us cognitive mechanisms at the origin of cultural biases. At the same time, however, we without a doubt concur with Laquer (1990) that “in the one-sex model, dominant in anatomical thinking for two thousand years, woman was understood as man inverted: the uterus was the female scrotum, the ovaries were testicles, the vulva was a foreskin, and *the vagina was a penis*” (p. 236, emphasis in the original). However, we want to distinguish a biological level, characterized by evolved decision-making processes for the solution of gender attribution problems, from a cultural and ethical level of the human sexuality that is not reducible to the constraints of cognitive bias. Therefore, we believe that the cultural stereotypes and prejudices that lead to sexual discrimination and oppression are not a mere and arbitrary cultural product, but the consequence of cognitive biases. This, far from justifying human behavior because it is biologically determined, must help us understand why, after millennia of civilization, gender discrimination resists cultural progress and gender equality movements (Konner, 2015). Gender equality requires historical remembrance and education on the salience of male sexual characteristics: They mediate gender attribution within milliseconds in the brain.

## Supplementary Information

Below is the link to the electronic supplementary material.

Supplementary file 1 (DOCX 1321 KB)
