# Supplementary material for: Sex/Gender Attribution: When the Penis Makes the Difference
Source: Arch Sex Behav. 2021 Nov 15;51(4):1865–79. doi: 10.1007/s10508-021-02152-z (PMC9192409; doi:10.1007/s10508-021-02152-z)
Supplement: Supplementary file 1 — (DOCX 1321 KB) [file 10508_2021_2152_MOESM1_ESM.docx]

Supplementary Material

to

Sex/Gender Attribution: When the Penis Makes the Difference

# Adult Gender Attribution Test

The 120 stimuli in the Adult Gender Attribution Test are shown below on the following page. The red framed stimuli 1_F1 (female) and 64_M1 (male) are the original images bought from the website [www.3d.sk](http://www.3d.sk) with a perpetual, non-exclusive, non-transferable worldwide license to use the Content for the Permitted Uses. All copyright and other intellectual property rights relating to the Content, are retained by 3D.sk. The other 118 stimuli not framed in read have been created through the software Adobe Photoshop 14 (see also Supplementary material).

**Table S1. The 120 stimuli of the Adult Gender Attribution Test.**

| 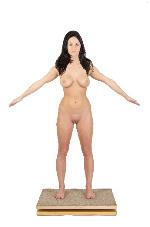 | 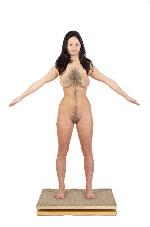 | 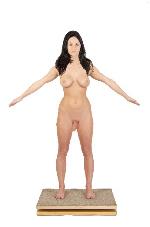 | 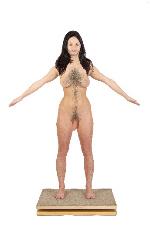 | 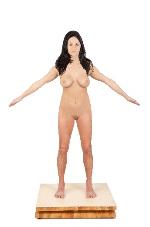 |
| --- | --- | --- | --- | --- |
| 1_F1 | 2_F2 | 3_F3 | 4_F8 | 5_F4 |
| 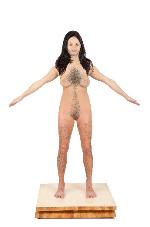 | 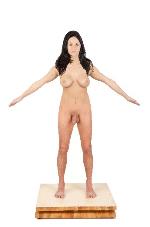 | 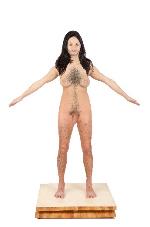 | 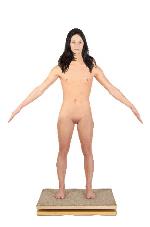 | 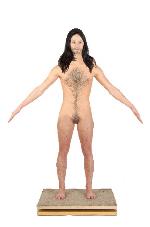 |
| 6_F9 | 7_F10 | 8_N1 | 9_F5 | 10_F11 |
| 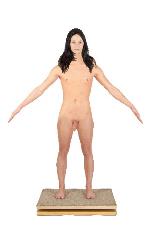 | 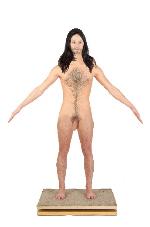 | 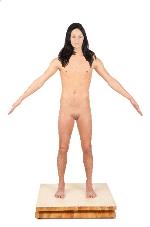 | 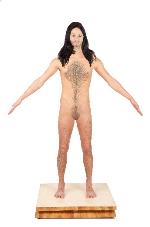 | 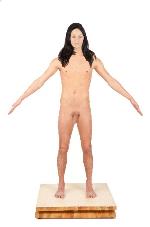 |
| 11_F12 | 12_N2 | 13_F13 | 14_N3 | 15_N4 |
| 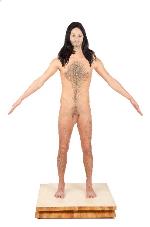 | 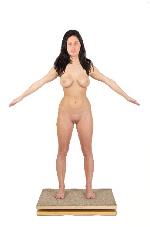 | 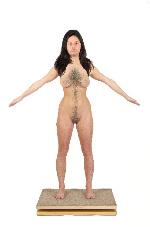 | 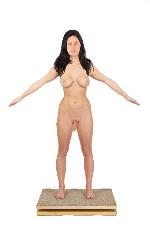 | 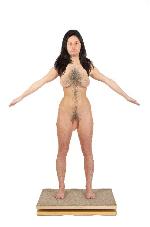 |
| 16_M8 | 17_F6 | 18_F14 | 19_F15 | 20_N5 |
| 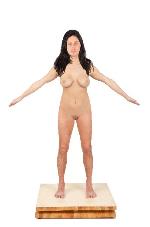 | 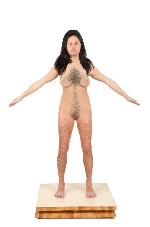 | 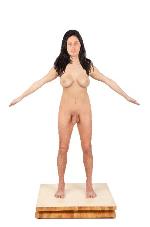 | 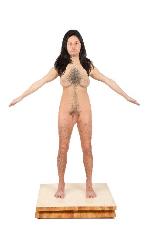 | 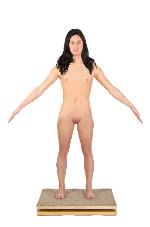 |
| 21_F16 | 22_N6 | 23_N7 | 24_M9 | 25_F17 |
| 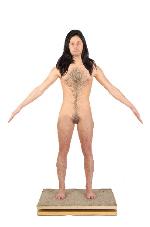 | 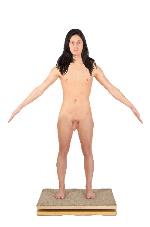 | 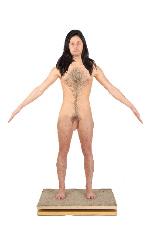 | 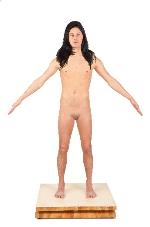 | 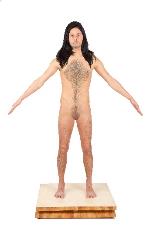 |
| 26_N8 | 27_N9 | 28_M10 | 29_N10 | 30_M11 |
| 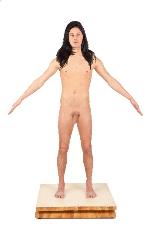 | 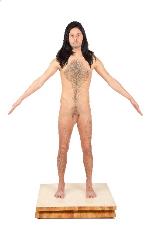 | 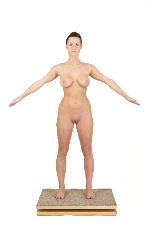 | 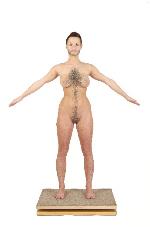 | 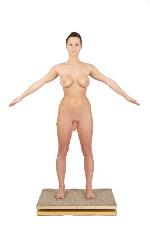 |
| 31_M12 | 32_M2 | 33_F7 | 34_F18 | 35_F19 |
| 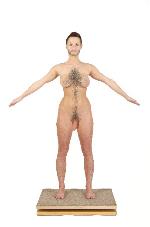 | 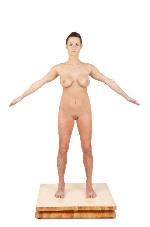 | 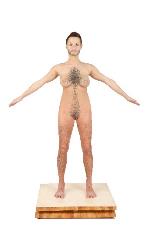 | 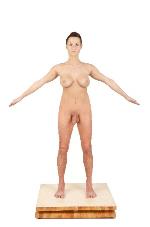 | 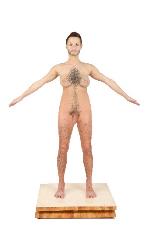 |
| 36_N11 | 37_F20 | 38_N12 | 39_N13 | 40_M13 |
| 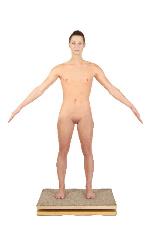 | 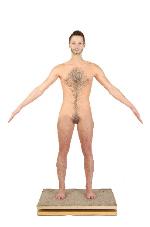 | 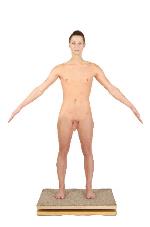 | 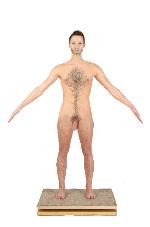 | 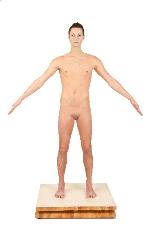 |
| 41_F21 | 42_N14 | 43_N15 | 44_M14 | 45_N16 |
| 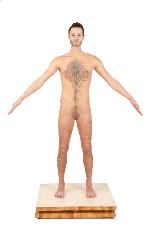 | 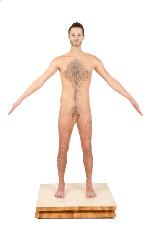 | 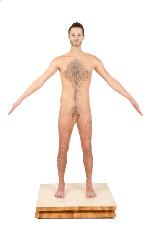 | 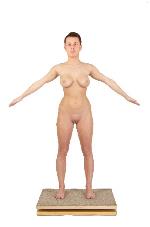 | 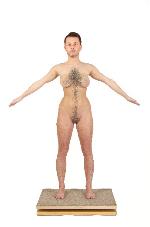 |
| 46_M15 | 47_M16 | 48_M3 | 49_F22 | 50_N17 |
| 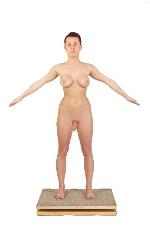 | 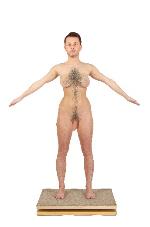 | 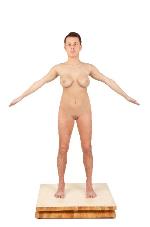 | 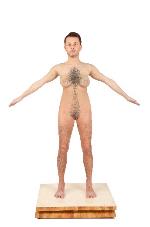 | 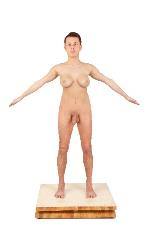 |
| 51_N18 | 52_M17 | 53_N19 | 54_M18 | 55_M19 |
| 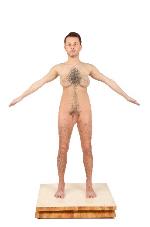 | 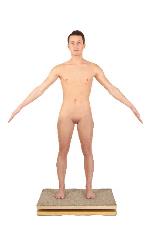 | 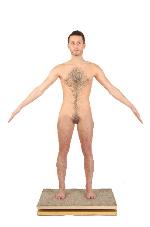 | 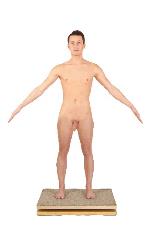 | 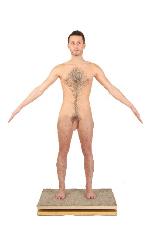 |
| 56_M4 | 57_N20 | 58_M20 | 59_M21 | 60_M5 |
| 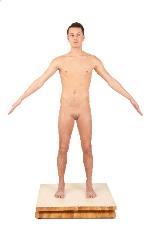 | 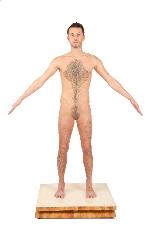 | 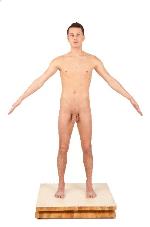 | 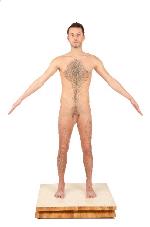 | 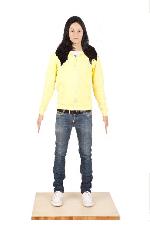 |
| 61_M22 | 62_M6 | 63_M7 | 64_M1 | 65_F69 |
| 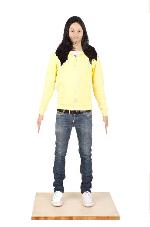 | 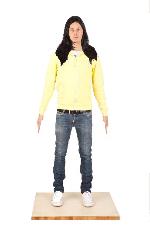 | 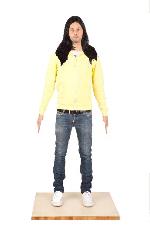 | 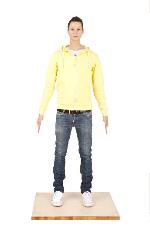 | 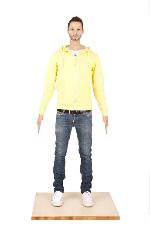 |
| 66_F70 | 67_F71 | 68_M66 | 69_F72 | 70_M67 |
| 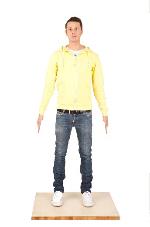 | 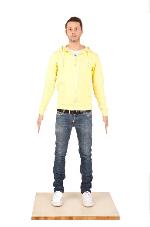 | 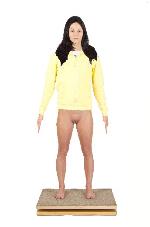 | 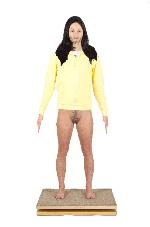 | 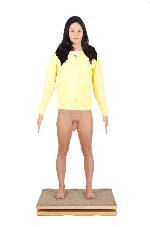 |
| 71_M68 | 72_M65 | 73_F89 | 74_F90 | 75_F91 |
| 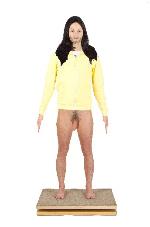 | 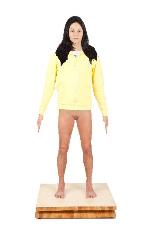 | 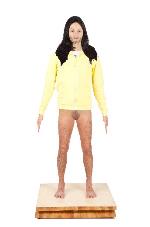 | 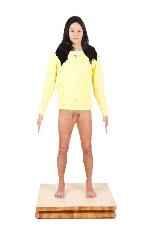 | 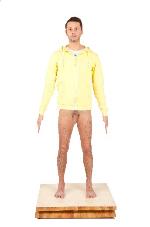 |
| 76_F95 | 77_F92 | 78_F96 | 79_F97 | 80_M73 |
| 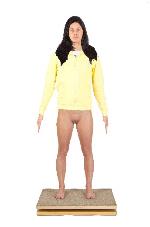 | 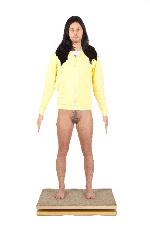 | 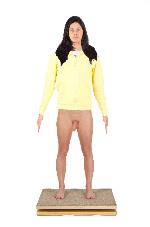 | 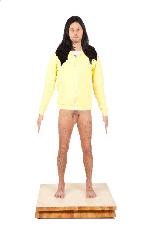 | 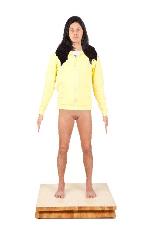 |
| 81_F93 | 82_F98 | 83_F99 | 84_M74 | 85_F100 |
| 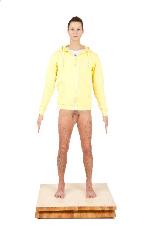 | 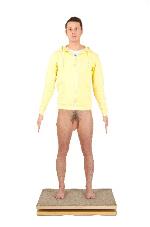 | 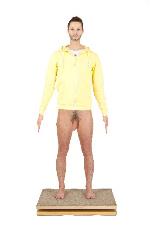 | 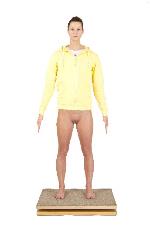 | 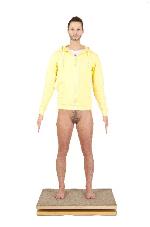 |
| 86_M75 | 87_M76 | 88_M83 | 89_F94 | 90_F101 |
| 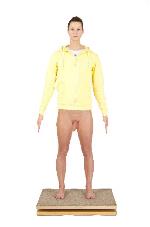 | 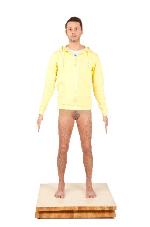 | 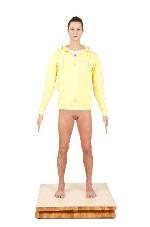 | 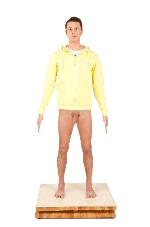 | 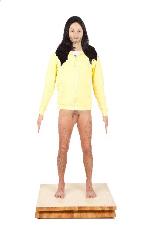 |
| 91_F102 | 92_M77 | 93_M103 | 94_M78 | 95_M79 |
| 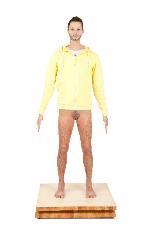 | 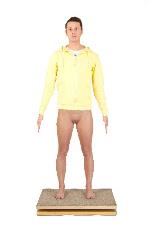 | 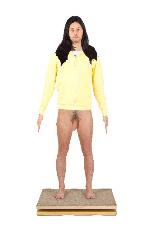 | 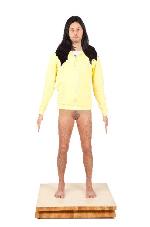 | 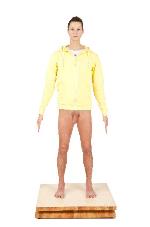 |
| 96_M84 | 97_F104 | 98_M80 | 99_M81 | 100_M85 |
|  |  |  |  |  |
| 101_M82 | 102_M86 | 103_M87 | 104_M88 | 105_F116 |
|  |  |  |  |  |
| 106_F117 | 107_F118 | 108_N105 | 109_F119 | 110_N106 |
|  |  |  |  |  |
| 111_N107 | 112_M112 | 113_F120 | 114_N108 | 115_N109 |
|  |  |  |  |  |
| 116_M113 | 117_N110 | 118_M114 | 119_M115 | 120_M111 |

**Table S2. Combination of twelve human physical characteristics (short hair, male face, flat chest, narrow hips, penis, body hair, long hair, female face, breast, wide hips, vulva, no body hair) and two cloths (t-shirt unisex, jeans unisex) variables for each image. In the column “Partial”, the number is the sum of occurrences of male variables. When the number is < 3 the image is considered feminine, = 3 the image is considered as neutral, > 3 the image is considered as a masculine. In the column “Total”, F = feminine, N = neutral, M = masculine. In the column “Codex”, the alphanumeric code refers to the image code as shown in the Table S1.**

| **Stimuli** | **Short hair** | **Male face** | **Flat chest** | **Narrow hips** | **Penis** | **Body hair** | **Partial** | **Long hair** | **Female face** | | **Breast** | | **Wide hips** | | **Vulva** | | **No body hair** | | **T-shirt unisex** | | **Jeans unisex** | | **Total** | | **Codex** | |
| --- | --- | --- | --- | --- | --- | --- | --- | --- | --- | --- | --- | --- | --- | --- | --- | --- | --- | --- | --- | --- | --- | --- | --- | --- | --- | --- |
| **1** | 0 | 0 | 0 | 0 | 0 | 0 | **0** | 1 | 1 | | 1 | | 1 | | 1 | | 1 | | 0 | | 0 | | **F** | | F1 | |
| **2** | 0 | 0 | 0 | 0 | 0 | 1 | **1** | 1 | 1 | | 1 | | 1 | | 1 | | 0 | | 0 | | 0 | | **F** | | F2 | |
| **3** | 0 | 0 | 0 | 0 | 1 | 0 | **1** | 1 | 1 | | 1 | | 1 | | 0 | | 1 | | 0 | | 0 | | **F** | | F3 | |
| **4** | 0 | 0 | 0 | 0 | 1 | 1 | **2** | 1 | 1 | | 1 | | 1 | | 0 | | 0 | | 0 | | 0 | | **F** | | F8 | |
| **5** | 0 | 0 | 0 | 1 | 0 | 0 | **1** | 1 | 1 | | 1 | | 0 | | 1 | | 1 | | 0 | | 0 | | **F** | | F4 | |
| **6** | 0 | 0 | 0 | 1 | 0 | 1 | **2** | 1 | 1 | | 1 | | 0 | | 1 | | 0 | | 0 | | 0 | | **F** | | F9 | |
| **7** | 0 | 0 | 0 | 1 | 1 | 0 | **2** | 1 | 1 | | 1 | | 0 | | 0 | | 1 | | 0 | | 0 | | **F** | | F10 | |
| **8** | 0 | 0 | 0 | 1 | 1 | 1 | **3** | 1 | 1 | | 1 | | 0 | | 0 | | 0 | | 0 | | 0 | | **N** | | N1 | |
| **9** | 0 | 0 | 1 | 0 | 0 | 0 | **1** | 1 | 1 | | 0 | | 1 | | 1 | | 1 | | 0 | | 0 | | **F** | | F5 | |
| **10** | 0 | 0 | 1 | 0 | 0 | 1 | **2** | 1 | 1 | | 0 | | 1 | | 1 | | 0 | | 0 | | 0 | | **F** | | F11 | |
| **11** | 0 | 0 | 1 | 0 | 1 | 0 | **2** | 1 | 1 | | 0 | | 1 | | 0 | | 1 | | 0 | | 0 | | **F** | | F12 | |
| **12** | 0 | 0 | 1 | 0 | 1 | 1 | **3** | 1 | 1 | | 0 | | 1 | | 0 | | 0 | | 0 | | 0 | | **N** | | N2 | |
| **13** | 0 | 0 | 1 | 1 | 0 | 0 | **2** | 1 | 1 | | 0 | | 0 | | 1 | | 1 | | 0 | | 0 | | **F** | | F13 | |
| **14** | 0 | 0 | 1 | 1 | 0 | 1 | **3** | 1 | 1 | | 0 | | 0 | | 1 | | 0 | | 0 | | 0 | | **N** | | N3 | |
| **15** | 0 | 0 | 1 | 1 | 1 | 0 | **3** | 1 | 1 | | 0 | | 0 | | 0 | | 1 | | 0 | | 0 | | **N** | | N4 | |
| **16** | 0 | 0 | 1 | 1 | 1 | 1 | **4** | 1 | 1 | | 0 | | 0 | | 0 | | 0 | | 0 | | 0 | | **M** | | M8 | |
| **17** | 0 | 1 | 0 | 0 | 0 | 0 | **1** | 1 | 0 | | 1 | | 1 | | 1 | | 1 | | 0 | | 0 | | **F** | | F6 | |
| **18** | 0 | 1 | 0 | 0 | 0 | 1 | **2** | 1 | 0 | | 1 | | 1 | | 1 | | 0 | | 0 | | 0 | | **F** | | F14 | |
| **19** | 0 | 1 | 0 | 0 | 1 | 0 | **2** | 1 | 0 | | 1 | | 1 | | 0 | | 1 | | 0 | | 0 | | **F** | | F15 | |
| **20** | 0 | 1 | 0 | 0 | 1 | 1 | **3** | 1 | 0 | | 1 | | 1 | | 0 | | 0 | | 0 | | 0 | | **N** | | N5 | |
| **21** | 0 | 1 | 0 | 1 | 0 | 0 | **2** | 1 | 0 | | 1 | | 0 | | 1 | | 1 | | 0 | | 0 | | **F** | | F16 | |
| **22** | 0 | 1 | 0 | 1 | 0 | 1 | **3** | 1 | 0 | | 1 | | 0 | | 1 | | 0 | | 0 | | 0 | | **N** | | N6 | |
| **23** | 0 | 1 | 0 | 1 | 1 | 0 | **3** | 1 | 0 | | 1 | | 0 | | 0 | | 1 | | 0 | | 0 | | **N** | | N7 | |
| **24** | 0 | 1 | 0 | 1 | 1 | 1 | **4** | 1 | 0 | | 1 | | 0 | | 0 | | 0 | | 0 | | 0 | | **M** | | M9 | |
| **25** | 0 | 1 | 1 | 0 | 0 | 0 | **2** | 1 | 0 | | 0 | | 1 | | 1 | | 1 | | 0 | | 0 | | **F** | | F17 | |
| **26** | 0 | 1 | 1 | 0 | 0 | 1 | **3** | 1 | 0 | | 0 | | 1 | | 1 | | 0 | | 0 | | 0 | | **N** | | N8 | |
| **27** | 0 | 1 | 1 | 0 | 1 | 0 | **3** | 1 | 0 | | 0 | | 1 | | 0 | | 1 | | 0 | | 0 | | **N** | | N9 | |
| **28** | 0 | 1 | 1 | 0 | 1 | 1 | **4** | 1 | 0 | | 0 | | 1 | | 0 | | 0 | | 0 | | 0 | | **M** | | M10 | |
| **29** | 0 | 1 | 1 | 1 | 0 | 0 | **3** | 1 | 0 | | 0 | | 0 | | 1 | | 1 | | 0 | | 0 | | **N** | | N10 | |
| **30** | 0 | 1 | 1 | 1 | 0 | 1 | **4** | 1 | 0 | | 0 | | 0 | | 1 | | 0 | | 0 | | 0 | | **M** | | M11 | |
| **31** | 0 | 1 | 1 | 1 | 1 | 0 | **4** | 1 | 0 | | 0 | | 0 | | 0 | | 1 | | 0 | | 0 | | **M** | | M12 | |
| **32** | 0 | 1 | 1 | 1 | 1 | 1 | **5** | 1 | 0 | | 0 | | 0 | | 0 | | 0 | | 0 | | 0 | | **M** | | M2 | |
| **33** | 1 | 0 | 0 | 0 | 0 | 0 | **1** | 0 | 1 | | 1 | | 1 | | 1 | | 1 | | 0 | | 0 | | **F** | | F7 | |
| **34** | 1 | 0 | 0 | 0 | 0 | 1 | **2** | 0 | 1 | | 1 | | 1 | | 1 | | 0 | | 0 | | 0 | | **F** | | F18 | |
| **35** | 1 | 0 | 0 | 0 | 1 | 0 | **2** | 0 | 1 | | 1 | | 1 | | 0 | | 1 | | 0 | | 0 | | **F** | | F19 | |
| **36** | 1 | 0 | 0 | 0 | 1 | 1 | **3** | 0 | 1 | | 1 | | 1 | | 0 | | 0 | | 0 | | 0 | | **N** | | N11 | |
| **37** | 1 | 0 | 0 | 1 | 0 | 0 | **2** | 0 | 1 | | 1 | | 0 | | 1 | | 1 | | 0 | | 0 | | **F** | | F20 | |
| **38** | 1 | 0 | 0 | 1 | 0 | 1 | **3** | 0 | 1 | | 1 | | 0 | | 1 | | 0 | | 0 | | 0 | | **N** | | N12 | |
| **39** | 1 | 0 | 0 | 1 | 1 | 0 | **3** | 0 | 1 | | 1 | | 0 | | 0 | | 1 | | 0 | | 0 | | **N** | | N13 | |
| **40** | 1 | 0 | 0 | 1 | 1 | 1 | **4** | 0 | 1 | | 1 | | 0 | | 0 | | 0 | | 0 | | 0 | | **M** | | M13 | |
| **41** | 1 | 0 | 1 | 0 | 0 | 0 | **2** | 0 | 1 | | 0 | | 1 | | 1 | | 1 | | 0 | | 0 | | **F** | | F21 | |
| **42** | 1 | 0 | 1 | 0 | 0 | 1 | **3** | 0 | 1 | | 0 | | 1 | | 1 | | 0 | | 0 | | 0 | | **N** | | N14 | |
| **43** | 1 | 0 | 1 | 0 | 1 | 0 | **3** | 0 | 1 | | 0 | | 1 | | 0 | | 1 | | 0 | | 0 | | **N** | | N15 | |
| **44** | 1 | 0 | 1 | 0 | 1 | 1 | **4** | 0 | 1 | | 0 | | 1 | | 0 | | 0 | | 0 | | 0 | | **M** | | M14 | |
| **45** | 1 | 0 | 1 | 1 | 0 | 0 | **3** | 0 | 1 | | 0 | | 0 | | 1 | | 1 | | 0 | | 0 | | **N** | | N16 | |
| **46** | 1 | 0 | 1 | 1 | 0 | 1 | **4** | 0 | 1 | | 0 | | 0 | | 1 | | 0 | | 0 | | 0 | | **M** | | M15 | |
| **47** | 1 | 0 | 1 | 1 | 1 | 0 | **4** | 0 | 1 | | 0 | | 0 | | 0 | | 1 | | 0 | | 0 | | **M** | | M16 | |
| **48** | 1 | 0 | 1 | 1 | 1 | 1 | **5** | 0 | 1 | | 0 | | 0 | | 0 | | 0 | | 0 | | 0 | | **M** | | M3 | |
| **49** | 1 | 1 | 0 | 0 | 0 | 0 | **2** | 0 | 0 | | 1 | | 1 | | 1 | | 1 | | 0 | | 0 | | **F** | | F22 | |
| **50** | 1 | 1 | 0 | 0 | 0 | 1 | **3** | 0 | 0 | | 1 | | 1 | | 1 | | 0 | | 0 | | 0 | | **N** | | N17 | |
| **51** | 1 | 1 | 0 | 0 | 1 | 0 | **3** | 0 | 0 | | 1 | | 1 | | 0 | | 1 | | 0 | | 0 | | **N** | | N18 | |
| **52** | 1 | 1 | 0 | 0 | 1 | 1 | **4** | 0 | 0 | | 1 | | 1 | | 0 | | 0 | | 0 | | 0 | | **M** | | M17 | |
| **53** | 1 | 1 | 0 | 1 | 0 | 0 | **3** | 0 | 0 | | 1 | | 0 | | 1 | | 1 | | 0 | | 0 | | **N** | | N19 | |
| **54** | 1 | 1 | 0 | 1 | 0 | 1 | **4** | 0 | 0 | | 1 | | 0 | | 1 | | 0 | | 0 | | 0 | | **M** | | M18 | |
| **55** | 1 | 1 | 0 | 1 | 1 | 0 | **4** | 0 | 0 | | 1 | | 0 | | 0 | | 1 | | 0 | | 0 | | **M** | | M19 | |
| **56** | 1 | 1 | 0 | 1 | 1 | 1 | **5** | 0 | 0 | | 1 | | 0 | | 0 | | 0 | | 0 | | 0 | | **M** | | M4 | |
| **57** | 1 | 1 | 1 | 0 | 0 | 0 | **3** | 0 | 0 | | 0 | | 1 | | 1 | | 1 | | 0 | | 0 | | **N** | | N20 | |
| **58** | 1 | 1 | 1 | 0 | 0 | 1 | **4** | 0 | 0 | | 0 | | 1 | | 1 | | 0 | | 0 | | 0 | | **M** | | M20 | |
| **59** | 1 | 1 | 1 | 0 | 1 | 0 | **4** | 0 | 0 | | 0 | | 1 | | 0 | | 1 | | 0 | | 0 | | **M** | | M21 | |
| **60** | 1 | 1 | 1 | 0 | 1 | 1 | **5** | 0 | 0 | | 0 | | 1 | | 0 | | 0 | | 0 | | 0 | | **M** | | M5 | |
| **61** | 1 | 1 | 1 | 1 | 0 | 0 | **4** | 0 | 0 | | 0 | | 0 | | 1 | | 1 | | 0 | | 0 | | **M** | | M22 | |
| **62** | 1 | 1 | 1 | 1 | 0 | 1 | **5** | 0 | 0 | | 0 | | 0 | | 1 | | 0 | | 0 | | 0 | | **M** | | M6 | |
| **63** | 1 | 1 | 1 | 1 | 1 | 0 | **5** | 0 | 0 | | 0 | | 0 | | 0 | | 1 | | 0 | | 0 | | **M** | | M7 | |
| **64** | 1 | 1 | 1 | 1 | 1 | 1 | **6** | 0 | 0 | | 0 | | 0 | | 0 | | 0 | | 0 | | 0 | | **M** | | M1 | |
| **65** | 0 | 0 | X | X | X | 0 | **0** | 1 | | 1 | | X | | X | | X | | 1 | | 1 | | 1 | | **F** | | F69 |
| **66** | 0 | 0 | X | X | X | 1 | **1** | 1 | | 1 | | X | | X | | X | | 0 | | 1 | | 1 | | **F** | | F70 |
| **67** | 0 | 1 | X | X | X | 0 | **1** | 1 | | 0 | | X | | X | | X | | 1 | | 1 | | 1 | | **F** | | F71 |
| **68** | 0 | 1 | X | X | X | 1 | **2** | 1 | | 0 | | X | | X | | X | | 0 | | 1 | | 1 | | **M** | | M66 |
| **69** | 1 | 0 | X | X | X | 0 | **1** | 0 | | 1 | | X | | X | | X | | 1 | | 1 | | 1 | | **F** | | F72 |
| **70** | 1 | 0 | X | X | X | 1 | **2** | 0 | | 1 | | X | | X | | X | | 0 | | 1 | | 1 | | **M** | | M67 |
| **71** | 1 | 1 | X | X | X | 0 | **2** | 0 | | 0 | | X | | X | | X | | 1 | | 1 | | 1 | | **M** | | M68 |
| **72** | 1 | 1 | X | X | X | 1 | **3** | 0 | | 0 | | X | | X | | X | | 0 | | 1 | | 1 | | **M** | | M65 |
| **73** | 0 | 0 | X | 0 | 0 | 0 | **0** | 1 | | 1 | | X | | 1 | | 1 | | 1 | | 1 | | 0 | | **F** | | F89 |
| **74** | 0 | 0 | X | 0 | 0 | 1 | **1** | 1 | | 1 | | X | | 1 | | 1 | | 0 | | 1 | | 0 | | **F** | | F90 |
| **75** | 0 | 0 | X | 0 | 1 | 0 | **1** | 1 | | 1 | | X | | 1 | | 0 | | 1 | | 1 | | 0 | | **F** | | F91 |
| **76** | 0 | 0 | X | 0 | 1 | 1 | **2** | 1 | | 1 | | X | | 1 | | 0 | | 0 | | 1 | | 0 | | **F** | | F95 |
| **77** | 0 | 0 | X | 1 | 0 | 0 | **1** | 1 | | 1 | | X | | 0 | | 1 | | 1 | | 1 | | 0 | | **F** | | F92 |
| **78** | 0 | 0 | X | 1 | 0 | 1 | **2** | 1 | | 1 | | X | | 0 | | 1 | | 0 | | 1 | | 0 | | **F** | | F96 |
| **79** | 0 | 0 | X | 1 | 1 | 0 | **2** | 1 | | 1 | | X | | 0 | | 0 | | 1 | | 1 | | 0 | | **F** | | F97 |
| **80** | 1 | 1 | X | 1 | 1 | 1 | **5** | 0 | | 0 | | X | | 0 | | 0 | | 0 | | 1 | | 0 | | **M** | | M73 |
| **81** | 0 | 1 | X | 0 | 0 | 0 | **1** | 1 | | 0 | | X | | 1 | | 1 | | 1 | | 1 | | 0 | | **F** | | F93 |
| **82** | 0 | 1 | X | 0 | 0 | 1 | **2** | 1 | | 0 | | X | | 1 | | 1 | | 0 | | 1 | | 0 | | **F** | | F98 |
| **83** | 0 | 1 | X | 0 | 1 | 0 | **2** | 1 | | 0 | | X | | 1 | | 0 | | 1 | | 1 | | 0 | | **F** | | F99 |
| **84** | 0 | 1 | X | 1 | 1 | 1 | **4** | 1 | | 0 | | X | | 0 | | 0 | | 0 | | 1 | | 0 | | **M** | | M74 |
| **85** | 0 | 1 | X | 1 | 0 | 0 | **2** | 1 | | 0 | | X | | 0 | | 1 | | 1 | | 1 | | 0 | | **F** | | F100 |
| **86** | 1 | 0 | X | 1 | 1 | 1 | **4** | 0 | | 1 | | X | | 0 | | 0 | | 0 | | 1 | | 0 | | **M** | | M75 |
| **87** | 1 | 1 | X | 0 | 1 | 1 | **4** | 0 | | 0 | | X | | 1 | | 0 | | 0 | | 1 | | 0 | | **M** | | M76 |
| **88** | 1 | 0 | X | 0 | 1 | 1 | **3** | 0 | | 1 | | X | | 1 | | 0 | | 0 | | 1 | | 0 | | **M** | | M83 |
| **89** | 1 | 0 | X | 0 | 0 | 0 | **1** | 0 | | 1 | | X | | 1 | | 1 | | 1 | | 1 | | 0 | | **F** | | F94 |
| **90** | 1 | 0 | X | 0 | 0 | 1 | **2** | 0 | | 1 | | X | | 1 | | 1 | | 0 | | 1 | | 0 | | **F** | | F101 |
| **91** | 1 | 0 | X | 0 | 1 | 0 | **2** | 0 | | 1 | | X | | 1 | | 0 | | 1 | | 1 | | 0 | | **F** | | F102 |
| **92** | 1 | 1 | X | 1 | 0 | 1 | **4** | 0 | | 0 | | X | | 0 | | 1 | | 0 | | 1 | | 0 | | **M** | | M77 |
| **93** | 1 | 0 | X | 1 | 0 | 0 | **2** | 0 | | 1 | | X | | 0 | | 1 | | 1 | | 1 | | 0 | | **F** | | F103 |
| **94** | 1 | 1 | X | 1 | 1 | 0 | **4** | 0 | | 0 | | X | | 0 | | 0 | | 1 | | 1 | | 0 | | **M** | | M78 |
| **95** | 0 | 0 | X | 1 | 1 | 1 | **3** | 1 | | 1 | | X | | 0 | | 0 | | 0 | | 1 | | 0 | | **M** | | M79 |
| **96** | 1 | 0 | X | 1 | 0 | 1 | **3** | 0 | | 1 | | X | | 0 | | 1 | | 0 | | 1 | | 0 | | **M** | | M84 |
| **97** | 1 | 1 | X | 0 | 0 | 0 | **2** | 0 | | 0 | | X | | 1 | | 1 | | 1 | | 1 | | 0 | | **F** | | F104 |
| **98** | 0 | 1 | X | 0 | 1 | 1 | **3** | 1 | | 0 | | X | | 1 | | 0 | | 0 | | 1 | | 0 | | **M** | | M80 |
| **99** | 0 | 1 | X | 1 | 0 | 1 | **3** | 1 | | 0 | | X | | 0 | | 1 | | 0 | | 1 | | 0 | | **M** | | M81 |
| **100** | 1 | 0 | X | 1 | 1 | 0 | **3** | 0 | | 1 | | X | | 0 | | 0 | | 1 | | 1 | | 0 | | **M** | | M85 |
| **101** | 0 | 1 | X | 1 | 1 | 0 | **3** | 1 | | 0 | | X | | 0 | | 0 | | 1 | | 1 | | 0 | | **M** | | M82 |
| **102** | 1 | 1 | X | 0 | 0 | 1 | **3** | 0 | | 0 | | X | | 1 | | 1 | | 0 | | 1 | | 0 | | **M** | | M86 |
| **103** | 1 | 1 | X | 0 | 1 | 0 | **3** | 0 | | 0 | | X | | 1 | | 0 | | 1 | | 1 | | 0 | | **M** | | M87 |
| **104** | 1 | 1 | X | 1 | 0 | 0 | **3** | 0 | | 0 | | X | | 0 | | 1 | | 1 | | 1 | | 0 | | **M** | | M88 |
| **105** | 0 | 0 | 0 | X | X | 0 | **0** | 1 | | 1 | | 1 | | X | | X | | 1 | | 0 | | 1 | | **F** | | F116 |
| **106** | 0 | 0 | 0 | X | X | 1 | **1** | 1 | | 1 | | 1 | | X | | X | | 0 | | 0 | | 1 | | **F** | | F117 |
| **107** | 0 | 0 | 1 | X | X | 0 | **1** | 1 | | 1 | | 0 | | X | | X | | 1 | | 0 | | 1 | | **F** | | F118 |
| **108** | 0 | 0 | 1 | X | X | 1 | **2** | 1 | | 1 | | 0 | | X | | X | | 0 | | 0 | | 1 | | **N** | | N105 |
| **109** | 0 | 1 | 0 | X | X | 0 | **1** | 1 | | 0 | | 1 | | X | | X | | 1 | | 0 | | 1 | | **F** | | F119 |
| **110** | 0 | 1 | 0 | X | X | 1 | **2** | 1 | | 0 | | 1 | | X | | X | | 0 | | 0 | | 1 | | **N** | | N106 |
| **111** | 0 | 1 | 1 | X | X | 0 | **2** | 1 | | 0 | | 0 | | X | | X | | 1 | | 0 | | 1 | | **N** | | N107 |
| **112** | 0 | 1 | 1 | X | X | 1 | **3** | 1 | | 0 | | 0 | | X | | X | | 0 | | 0 | | 1 | | **M** | | M112 |
| **113** | 1 | 0 | 0 | X | X | 0 | **1** | 0 | | 1 | | 1 | | X | | X | | 1 | | 0 | | 1 | | **F** | | F120 |
| **114** | 1 | 0 | 0 | X | X | 1 | **2** | 0 | | 1 | | 1 | | X | | X | | 0 | | 0 | | 1 | | **N** | | N108 |
| **115** | 1 | 0 | 1 | X | X | 0 | **2** | 0 | | 1 | | 0 | | X | | X | | 1 | | 0 | | 1 | | **N** | | N109 |
| **116** | 1 | 0 | 1 | X | X | 1 | **3** | 0 | | 1 | | 0 | | X | | X | | 0 | | 0 | | 1 | | **M** | | M113 |
| **117** | 1 | 1 | 0 | X | X | 0 | **2** | 0 | | 0 | | 1 | | X | | X | | 1 | | 0 | | 1 | | **N** | | N110 |
| **118** | 1 | 1 | 0 | X | X | 1 | **3** | 0 | | 0 | | 1 | | X | | X | | 0 | | 0 | | 1 | | **M** | | M114 |
| **119** | 1 | 1 | 1 | X | X | 0 | **3** | 0 | | 0 | | 0 | | X | | X | | 1 | | 0 | | 1 | | **M** | | M115 |
| **120** | 1 | 1 | 1 | X | X | 1 | **4** | 0 | | 0 | | 0 | | X | | X | | 0 | | 0 | | 1 | | **M** | | M111 |

# Participants’ description

A *t*-test was run where the participant’s gender in a binary mode (man/woman) was the independent variable and the dependent were the means of the responses to the Adult Gender Attribution Test (Table S3).

**Table S3. T-test on sexual orientation over biological sex.**

|  | Levene’test for quality of variance | t-test for Equality of Means |  |  |  |  |  |  |  |
| --- | --- | --- | --- | --- | --- | --- | --- | --- | --- |
|  | *F* | *p.* | *t* | gl | Sign. (2-tailes) | Mean Difference | Std. Error Difference | 95% Confidence  Interval of the Difference | |
|  |  |  |  |  |  |  |  | Lower | Upper |
| Equal variances (presumed) | 7.406 | 0.007 | 1.241 | 590 | 0.215 | 0.123 | 0.099 | -0.072 | 0.317 |
| Equal variances (not presumed) |  |  | 1.126 | 289.661 | 0.261 | 0.123 | 0.109 | -0.092 | 0.338 |

A one-way between-subjects ANOVAs were conducted to compare the effect of the independent variables—extracted through the Sociodemographic questionnaire and the Kinsey scale (sexual orientation)—on the means of the gender attribution (Tables: S4, S5, S6, S7, S8).

**Table S4. One-way ANOVA: gender attribution (mean) over sexual identity (three categories: male, female, other).**

|  | Sum of squares | df | Mean (squared) | *F* | *p.* |
| --- | --- | --- | --- | --- | --- |
| Between groups | 0.008 | 2 | 0.004 | 0.497 | 0.609 |
| Within groups | 4.546 | 589 | 0.008 |  |  |
| Total | 4.554 | 591 |  |  |  |

**Table S5. One-way ANOVA: gender attribution (mean) over sexual orientation (three categories: heterosexual, bisexual and homosexual).**

|  | Sum of squares | df | Mean (squared) | *F* | *p.* |
| --- | --- | --- | --- | --- | --- |
| Between groups | 0.003 | 2 | 0.001 | 0.180 | 0.836 |
| Within groups | 4.551 | 589 | 0.008 |  |  |
| Total | 4.554 | 591 |  |  |  |

**Table S6. One-way ANOVA: gender attribution (mean) over religion (three categories: Catholic, no religion and other).**

|  | Sum of squares | df | Mean (squared) | *F* | *p.* |
| --- | --- | --- | --- | --- | --- |
| Between groups | 0.028 | 2 | 0.014 | 1.816 | 0.164 |
| Within groups | 4.526 | 589 | 0.008 |  |  |
| Total | 4.554 | 591 |  |  |  |

**Table S7. One-way ANOVA: gender attribution (mean) over political orientation (five categories: Right-wing, Centre, Left-wing, none, Doesn’t know)**

|  | Sum of squares | df | Mean (squared) | *F* | *p.* |
| --- | --- | --- | --- | --- | --- |
| Between groups | 0.047 | 4 | 0.012 | 1.533 | 0.191 |
| Within groups | 4.507 | 587 | 0.008 |  |  |
| Total | 4.554 | 591 |  |  |  |

**Table S8. One-way ANOVA: gender attribution (mean) over level of education (five categories: Secondary school diploma, Secondary school higher diploma, degree, master degree, post-graduate)**

|  | Sum of squares | df | Mean (squared) | *F* | *p.* |
| --- | --- | --- | --- | --- | --- |
| Between groups | 0.004 | 3 | 0.001 | 0.165 | 0.920 |
| Within groups | 4.550 | 588 | 0.008 |  |  |
| Total | 4.554 | 591 |  |  |  |

**Table S9. Cronbach’s alpha: gender attribution for the 120 stimuli**

| Case Processing Summary | | | |
| --- | --- | --- | --- |
|  | | N | % |
| Cases | Valid | 581 | 98.1 |
|  | Excluded^a^ | 11 | 1.9 |
|  | Total | 592 | 100.0 |
| a. Listwise deletion based on all variables in the procedure. | | | |

| Reliability Statistics | | |
| --- | --- | --- |
| Cronbach’s Alpha | Cronbach’s Alpha Based on Standardized Items | N of Items |
| .855 | .826 | 120 |

**Table S10. Cronbach’s alpha: confidence scale for the 120 stimuli**

| Case Processing Summary | | | |
| --- | --- | --- | --- |
|  | | N | % |
| Cases | Valid | 592 | 100.0 |
|  | Excluded^a^ | 0 | .0 |
|  | Total | 592 | 100.0 |
| a. Listwise deletion based on all variables in the procedure. | | | |

| Reliability Statistics | | |
| --- | --- | --- |
| Cronbach’s Alpha | Cronbach’s Alpha Based on Standardized Items | N of Items |
| .992 | .992 | 120 |

**Table S11. Cronbach’s alpha: pleasantness scale for the 120 stimuli.**

| Case Processing Summary | | | |
| --- | --- | --- | --- |
|  | | N | % |
| Cases | Valid | 581 | 98.1 |
|  | Excluded^a^ | 11 | 1.9 |
|  | Total | 592 | 100.0 |
| a. Listwise deletion based on all variables in the procedure. | | | |

| Reliability Statistics | | |
| --- | --- | --- |
| Cronbach’s Alpha | Cronbach’s Alpha Based on Standardized Items | N of Items |
| .992 | .992 | 120 |

# The male sexual characteristics effect on gender attribution (H1.a)

- See “H1a_mixedlogistic.spv” in “Data Sheet and SPSS Regression Outputs.zip” (Federici et al., 2020),

# Primary sexual characteristics on gender attribution (H1.b)

- See “H1b e H1d_mixedlogistic.spv” in “Data Sheet and SPSS Regression Outputs.zip” (Federici et al., 2020),

# Primary sexual characteristics (H1.c)

- See “H1c e H2_mixedlogistic.spv” in “Data Sheet and SPSS Regression Outputs.zip” (Federici et al., 2020),

# The penis effect on gender attribution (H1.d)

- See “H1b e H1d_mixedlogistic.spv” in “Data Sheet and SPSS Regression Outputs.zip” (Federici et al., 2020),

# Face effect on gender attribution (H2)

- See “H1c e H2_mixedlogistic.spv” in “Data Sheet and SPSS Regression Outputs.zip” (Federici et al., 2020).

# Pre-experimental phase

In the following table are shown the twenty human models proposed for the choice of the two “original” images on which the 120 stimuli of the Adult Gender Attribution Test were depicted. The twenty photographs were bought from the website [www.3d.sk](http://www.3d.sk); all had the same digital visual characteristics (light, exposure time, distance from the lens, resolution, etc.) across male and female models; all were shown in full nude in frontal position, aged between 21 and 30 years, white Caucasian, without visible marks on the body such as scars or tattoos.

**Table S12. The 20 human images, male and female, administered in the pre-experimental phase. The two red framed images are the those that received the highest preference score as either more masculine or more feminine and adopt as “original” images for the Adult Gender Attribution Test (see above Table S1, stimuli 1_F1 and 64_M1).**

|  |  |
| --- | --- |
|  |  |
|  |  |
|  |  |
|  |  |
|  |  |
|  |  |
|  |  |
|  |  |
|  |  |

**References**

Federici, S., Lepri, A., & D’Urzo, E. (2020). Adult gender attribution test data: Raw data and spss regression outputs. *ResearchGate*. <https://doi.org/10.13140/RG.2.2.30722.91840>
